# Marine macroalgae-associated fungi from Yacila and Los Cangrejos beaches (Northern Peru) and previously selected marine bacteria evaluated as plant growth promoters in maize under saline stress

**DOI:** 10.3389/ffunb.2025.1726850

**Published:** 2026-01-14

**Authors:** Liset Milagros Arcela-Castro, Danay Betsabe Morales-Medina, Miriam Marleni Rosales-Cuentas, Carlos Augusto Salazar-Sandoval, Heber Peleg Cornelio-Santiago, Jube Ciro Portalatino-Zevallos, Edwin Jorge Vega-Portalatino

**Affiliations:** 1 Laboratorio de Biotecnología Microbiana, Universidad Nacional de Frontera, Sullana, Piura, Peru; 2 Laboratorio de Análisis de Agua y Suelo, Universidad Nacional de Frontera, Sullana, Piura, Peru; 3 Facultad de Ciencias de los Alimentos, Universidad Le Cordon Bleu, Magdalena del Mar, Lima, Peru; 4 Facultad de Ciencias, Universidad Nacional Santiago Antúnez de Mayolo, Huaraz, Ancash, Peru

**Keywords:** corn, endophytes, epiphytic, plant-growth, salt-stress

## Abstract

The diversity of marine fungi associated with macroalgae in Peru remains largely unknown, and no studies have provided holistic data on their biodiversity or their role as plant growth promoters in maize under salinity stress conditions. Endophytic and epiphytic fungi were isolated from the macroalgae *Caulerpa* sp., *Ulva* sp., *Ahnfeltiopsis* sp., and *Chondracanthus chamissoi*, collected from Yacila and Cangrejos beaches (Piura, Peru), while marine bacteria were provided by the Microbial Biotechnology Research Laboratory of the National University of Frontera. The growth-promoting properties of these marine microorganisms were evaluated prior to their inoculation in maize. Fungal isolates were phylogenetically characterized by ITS sequencing as *Penicillium* sp. YAFL13, *Penicillium* sp. YUFE7, *Talaromyces stollii* YAFL19, *T. stollii* YAFL4, *T. amestolkiae* YCFR3, *Aspergillus sydowii* CCDF2, *A. sydowii* YFep2, and *A. sydowii* YFep3. In total, 12 marine fungi were isolated and used in the assays along with 10 marine bacteria. Based on antimicrobial activity, IAA synthesis, siderophore production, phosphate solubilization, and hydrolytic enzyme production, three fungal strains (*Penicillium* sp. YAFL13, *A. sydowii* CCDF2, and *A. sydowii* YFep2) and two bacterial strains (*Bacillus* sp. YCFR5 and *Pantoea agglomerans* YAFL6) were selected. Among them, *A. sydowii* CCDF2 significantly enhanced maize growth parameters, highlighting its promising potential as a plant inoculant. This study represents one of the few reports on marine microorganisms associated with marine macroalgae, revealing a valuable fungal diversity and its potential role in promoting maize growth under saline stress conditions.

## 1 Introduction

Soil salinization, intensified by climate change and inadequate agricultural practices, affects more than one billion hectares worldwide and represents a serious threat to food security, according to the Food and Agriculture Organization of the United Nations (Liu et al., 2025b). Salinity stress limits the growth and yield of crops such as maize (*Zea mays* L.), particularly in arid and semi-arid regions (Doğru, 2021), by inducing ethylene accumulation, which inhibits plant development. In contrast, polyamine synthesis activates physiological responses that improve stress tolerance (El-Tarabily et al., 2020, 2021). Due to the limited effectiveness of conventional strategies, such as genetic improvement, interest in the use of plant growth-promoting microorganisms has increased as a sustainable alternative to mitigate the effects of salinity stress.

Plant growth-promoting rhizobacteria (PGPR) and endophytic fungi have demonstrated multiple beneficial mechanisms of action, including phosphate solubilization, nitrogen fixation, iron acquisition, and the synthesis of phytohormones such as auxins, gibberellins, cytokinins, ethylene, and abscisic acid (El-Tarabily et al., 2021; Patel et al., 2023; Watts et al., 2023). Additionally, many of these microorganisms enhance stress tolerance by reducing the levels of 1-aminocyclopropane-1-carboxylic acid (ACC), the precursor of ethylene, through the activity of ACC deaminase, thereby decreasing ethylene accumulation and promoting greater growth under saline conditions (Acuña et al., 2019).

Although these effects have been widely studied in terrestrial microorganisms, marine microorganisms have received considerably less attention (Rathod et al., 2023). The marine environment presents extreme conditions of salinity, pressure, and temperature, which act as selective factors driving the evolution of unique adaptive mechanisms in bacteria and fungi (Kumar et al., 2022; Behera and Das, 2023). These adaptations include the production of bioactive metabolites such as flavonoids, sterols, tannins, and polysaccharides, which not only support microbial survival but may also be exploited in agricultural applications (Hagaggi and Abdul-Raouf, 2022; Rathod et al., 2023).

Marine macroalgae represent important natural hosts for these microorganisms and are primarily classified as brown algae (Phaeophyceae, ~1,755 species), red algae (Rhodophyta, ~6,000 species), and green algae (Chlorophyta, ~1,500 species) (Abdelrazek et al., 2024). These algae play a crucial role in marine primary production by generating nutrient-rich habitats that facilitate colonization by diverse organisms (Vega-portalatino et al., 2024). Moreover, they are a source of bioactive compounds with potential pharmacological, food, and agricultural applications (Latif et al., 2022; Delgado et al., 2021; Cuchca Ramos et al., 2025).

On the Peruvian coast, species such as *Chondracanthus chamissoi* (“yuyo”), *Caulerpa* sp., *Ulva* sp., and *Ahnfeltiopsis* sp. have been identified as macroalgae of interest. *Chondracanthus chamissoi*, a red alga distributed in Peru and Chile, has high commercial value and is used for carrageenan extraction and as an organic fertilizer (Arbaiza et al., 2023; Diaz Ruíz et al., 2021; Soto-Deza et al., 2025). *Caulerpa* sp., although reported to have bactericidal activity (Santa M. et al., 2023), has no known agricultural applications (Aguilar et al., 2022). *Ulva* sp., on the other hand, has shown biostimulant effects in crops such as *Lactuca sativa* (Cuchca Ramos et al., 2025), while *Ahnfeltiopsis* sp., also found on the northern coast of Peru, has not yet been studied for agricultural use (Vega-portalatino et al., 2024).

One of the challenges in using macroalgae directly as biostimulants is the requirement of large volumes of biomass to obtain small amounts of active metabolites. In this regard, endophytic microorganisms associated with macroalgae represent a more viable alternative, as they can be cultivated under laboratory conditions for large-scale production of bioactive compounds (Wong Chin et al., 2021). However, the microbial biodiversity of these associations remains poorly understood.

The composition of endophytic communities in macroalgae varies considerably depending on multiple factors, including algal species and genotype, tissue origin, phenological stage, nutrient availability, geographic location, and host interaction (Dastogeer et al., 2018; Wong Chin et al., 2021). This complexity has driven increasing interest in exploring unique marine fungal taxa, many of which produce metabolites with potentially beneficial functions for plant growth under abiotic stress conditions (El-Bondkly et al., 2021; Hagaggi and Abdul-Raouf, 2022).

In addition, bacterial and fungal cultures offer advantages such as efficient biomass production, resistance to industrial conditions (agitation, pressure), and precise control of variables such as pH, temperature, and culture time, making them ideal candidates for large-scale biotechnological applications (Vasanthkumar et al., 2024). In line with these considerations, in this study, marine fungi were isolated from macroalgae collected from the beaches of Yacila and Cangrejos (Piura, Peru) with the objective of evaluating their plant growth-promoting capacity through antimicrobial activity assays, IAA synthesis, siderophore production, phosphate solubilization, and the production of hydrolytic enzymes such as cellulases, pectinases, proteases, and amylases, along with marine endophytic bacteria. Furthermore, phylogenetic identification was performed using molecular analyses on marine fungi preselected for their biostimulant activity. Finally, the effects of fungi and bacteria on maize growth under saline stress conditions were evaluated, with the aim of developing effective microbial formulations and highlighting the potential of these microorganisms for sustainable agriculture.

## 2 Materials and methods

### 2.1 Collection of macroalgae and isolation of endophytic and epiphytic fungus

**Figure 1:**
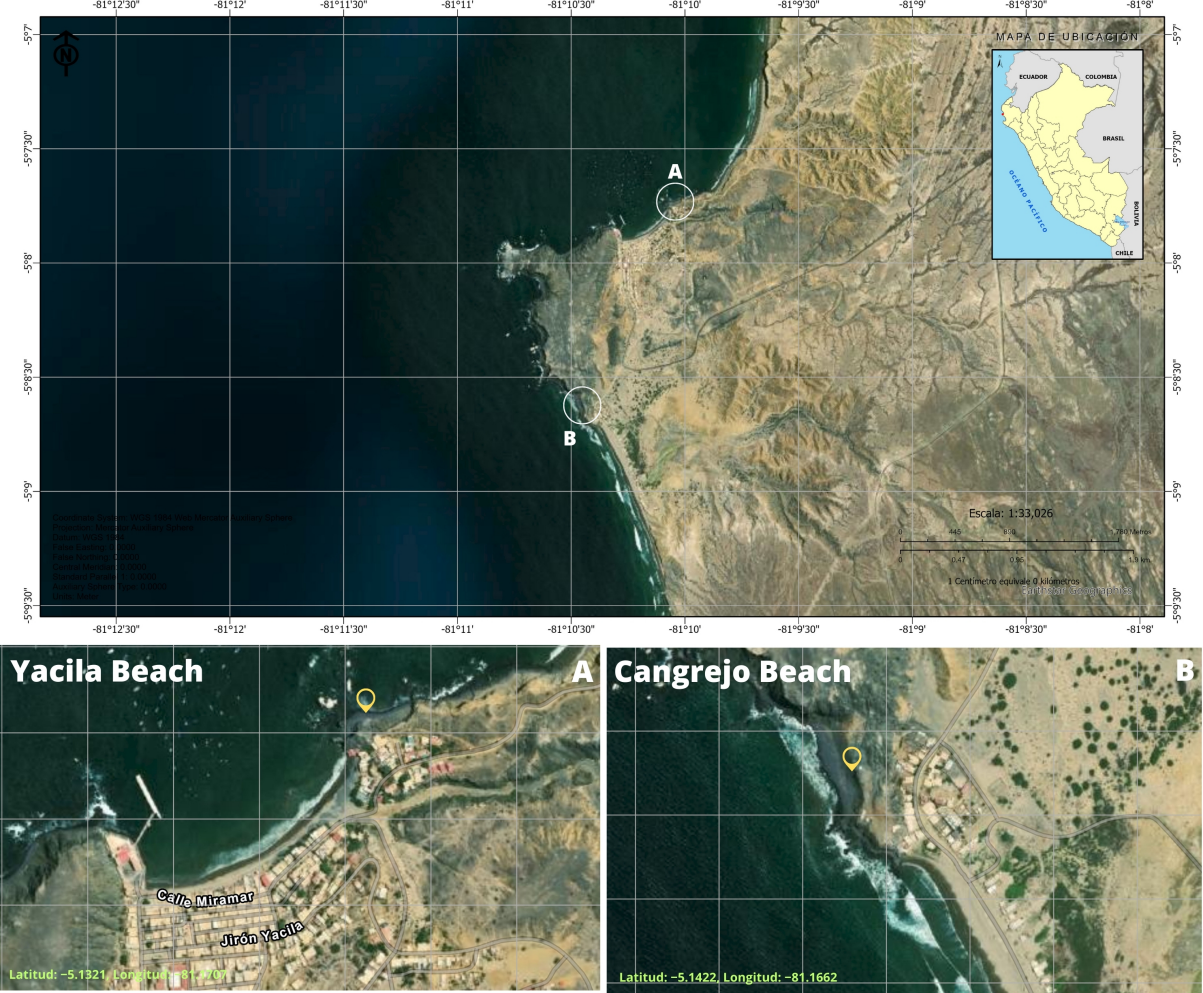
Collection sites of marine macroalgae (Northern coast of Peru). **(A)** Yacila Beach and **(B)** Los Cangrejos Beach. Source: Own elaboration (Software: National Geographic Institute of Peru – Center for Geospatial Data Infrastructure).

Macroalgae samples were collected in March 2024 from two coastal sites in the Piura region, northern Peru: Yacila Beach (Latitude: −5.1321, Longitude: −81.1707) and Los Cangrejos Beach (Latitude: −5.1422, Longitude: −81.1662), both located in the province of Paita (**Figure 1**). Four macroalgae species were collected: *Caulerpa* sp., *Ahnfeltiopsis* sp., *Ulva* sp., and *Chondracanthus chamissoi*, taxonomically identified by our research group, and endophytic and epiphytic fungi were isolated following the methodology by Vega-portalatino et al. (2024) with some modifications. In this case, macroalgae fragments were transferred to potato dextrose agar (PDA) supplemented with chloramphenicol (200 mg/mL) and incubated at 25 °C for 7 to 10 days. A fungus was considered endophytic when the control vial containing 2 mL of potato dextrose broth (PDB) showed no microbial growth. Subsequently, fungi growing on the fragments were purified by point inoculation on Petri dishes with PDA. Regarding epiphytic, *Caulerpa* sp. fragments were washed in tubes containing saline solution (NaCl 0.8%) at a 1/10 (w/v) ratio and inoculated by spread plating with sterile swabs on Petri dishes containing PDA, then purified in the same manner. Marine fungi (endophytes and epiphytic) were preserved in slant tubes with PDA. Additionally, all spores of axenic fungi were cryopreserved in cryovials using a base solution of 30% glycerol, 0.05% agar-agar, and 0.05% Tween 20, according to (Ulloa-Muñoz et al., 2020).

In addition, physicochemical parameters of the water were measured (**Table 1**), including pH, temperature, total dissolved solids, dissolved oxygen, atmospheric pressure, electrical conductivity, and turbidity, using a multiparameter device (HANNA, model HI98194, Romania) at the macroalgae collection sites on Yacila and Los Cangrejos beaches.

**Table 1:** Physicochemical parameters of the water at the macroalgae collection sites on Yacila and Los Cangrejos beaches.

Puntos de colecta de las macroalgas	pH	T (°C)	STD (mg/L)	OD (mg/L)	PSI	Ce (mS/cm)	Tu (NTU)
Cangrejos Beach	7.84	22.83	119	490	14.74	116.6	0.4
Yacila Beach	7.65	22.59	171	500	14.72	60.9	0.5

pH, hydrogen potential; T, temperature (°C); TDS, total dissolved solids (mg/L); DO, dissolved oxygen (mg/L); PSI, atmospheric pressure; EC, electrical conductivity (mS/cm), and Tu, turbidity (NTU).

### 2.2 Reactivation of marine endophytic bacteria

Marine endophytic bacteria, including *Kluyvera ascorbata* YAFL9 (PP328489.1), *K. ascorbata* YAFE21 (PP327419.1), *Enterobacter* sp. YAFL1 (PP328529.2), *Pantoea agglomerans* YAFL6 (PP328531.1), *Leclercia adecarboxylata* CUFE2 (PP328544.1), and four *Bacillus* sp. strains YCFE4 (PP329524.2), YUFE8 (PP329010.2), YCFR6 (PP329304.2), and YCFR5 (PP329066.2), as well as *Microbacterium paraoxydans* YCFE1 (PP329309.1) (Vega-portalatino et al., 2024), which were isolated by our research group at the Microbial Biotechnology Research Laboratory of the National University of Frontera, were inoculated into slant tubes containing Trypticase Soy Agar (TSA).

### 2.3 Plant growth promotion assays of marine bacteria and fungi

For endophytic and pathogenic bacteria, fresh 16-hour cultures were diluted with 0.8% NaCl to an optical density (OD_620nm_) of 0.08. For marine and pathogenic fungi, 5 mm discs were cut from PDA medium in Petri dishes after 5 days of incubation and used as inoculum for the different assays.

#### 2.3.1 Antibacterial activity

Marine fungi were tested against Gram-positive pathogenic bacteria (*Enterococcus faecalis* ATCC29212, *Staphylococcus epidermidis* ATCC12228, *Staphylococcus aureus* ATCC25923, S. aureus ATCC29213, *S. aureus* ATCC700699, and *Listeria monocytogenes* ATCC7644) and Gram-negative bacteria (*Escherichia coli* O157:H7, *E. coli* ATCC10536, and *Salmonella enterica* sv. *Typhimurium* ATCC14028).

These marine fungi were streaked on Petri plates containing PDA, after which 7 mL of molten PDA medium was dispensed and the plates were incubated at 25 °C for 7 days to allow the production of antibacterial metabolites. The cultures were then cut into 5 mm discs for testing using the Over-Plate Test (OpT) (Vega-portalatino et al., 2024). Pathogenic bacteria were grown in 4 mL of Trypticase Soy Broth (TSB) at 37 °C for 24 h and adjusted to an optical density (OD_620_nm) of 0.08. The bacterial suspension was then spread evenly over the surface of PDA plates. Immediately afterward, three fungal discs (5 mm) were placed onto the plates containing the pathogens. A 5 mm PDA disc was used as the negative control. Clear halos around the discs indicated antibacterial activity and were measured in millimeters (mm).

#### 2.3.2 Anticandidal activity

Marine fungi were tested against *Candida albicans* ATCC90028 and *Candida tropicalis* ATCC750 using the Over-Plate Test (OpT) (Vega-portalatino et al., 2024) technique on potato dextrose agar (PDA).

#### 2.3.3 Antifungal activity

The filamentous fungi used were *Fusarium* sp. H, *F. oxysporum* CTLM12 and *Colletotrichum* sp (Vega-portalatino et al., 2024), which were tested using the dual culture technique with some modifications by Nawrot-Chorabik et al. (2021). For marine fungi, 5 mm mycelial discs were placed in each corner of Petri plates containing PDA, and immediately afterward, a 5 mm disc of the filamentous fungus was placed in the center of the plate. The plates were then incubated at 25 °C for 3 to 10 days. For endophytic bacteria, 2 µL of fresh culture (OD_620_nm: 0.08) was inoculated onto four 6 mm filter paper discs, which were positioned following the procedure described above. PDA plates without endophytic microorganisms served as growth controls. The inhibitory capacity was determined by calculating the percentage of fungal inhibition (Ifo%) = [(A − B)/A] × 100, where A is the diameter of the pathogen without the endophytic fungus, and B is the diameter of the pathogen when interacting with the endophytic fungus (Ulloa-Muñoz et al., 2020).

#### 2.3.4 Indole-3-acetic acid production

The synthesis of IAA by endophytic bacteria and marine fungi was evaluated following the methodology described by Ulloa-Muñoz et al. (2020), with slight modifications. For marine fungi, one mycelial disc (5 mm) was inoculated into cryovials containing 1.5 mL of PDB supplemented with L-tryptophan (2 mg/mL) and NaCl (2% and 5%), and incubated at 25 °C for seven days. For endophytic bacteria, 75 µL of each bacterial suspension (OD_620nm_= 0.08) was inoculated into 1.5 mL of Tryptic Soy Broth (TSB) supplemented with L-tryptophan (2 mg/mL) and NaCl (2% and 5%), and incubated at 25 °C for 48 hours. After incubation, bacterial and fungal cells were removed by centrifugation at 2000 × g for four minutes. Next, 100 µL of the resulting supernatant was transferred to each well of a 96-well microplate containing 100 µL of Salkowski reagent. The plate was kept in the dark for 30 minutes. A positive reaction was indicated by the development of a pink color. All samples were prepared in triplicate. Based on preliminary assays, three bacterial strains (*Enterobacter* sp. YAFL1, *Bacillus* sp. YCFE4, and *Pantoea agglomerans* YAFL6) and two endophytic fungi (L3 and C21) were selected for IAA quantification using a UV–Vis spectrophotometer at 530 nm. A standard calibration curve was prepared using IAA concentrations of 0, 10, 20, 30, 40, 50, 60, and 70 µg/mL (Sigma) diluted in TSB (Ulloa-Muñoz et al., 2020).

#### 2.3.5 Siderophore production

To evaluate siderophore synthesis ability, Petri dishes were prepared containing one half Chrome Azurol S (CAS) medium and the other half Tryptic Soy Agar (TSA) or Potato Dextrose Agar (PDA) (Ulloa-Muñoz et al., 2020), supplemented with NaCl (2% and 5%). For marine fungi, two mycelial discs (5 mm) were placed at the interface between the CAS and PDA sections of the plate and incubated at 25°C for ten days. For endophytic bacteria, 2 µL of each bacterial suspension (OD_620_ = 0.08) was inoculated onto two sterile filter paper discs (6 mm), which were then placed at the interface between the CAS and TSA sections and incubated at 25°C for seven days. As negative controls, filter paper discs containing NaCl (0.8%) were used for bacteria, and PDA agar discs were used for fungi. Siderophore production was considered positive when the CAS medium changed color from blue to yellow, reddish, or purple. The reaction intensity was evaluated based on the degree of color change.

#### 2.3.6 Phosphate solubilization

Phosphate solubilization was evaluated following the methodology described by Ulloa-Muñoz et al. (2020). For marine fungi, three mycelial discs (5 mm) were placed on Petri dishes containing NBRIP medium supplemented with Ca_3_PO_4_ and NaCl (2% and 5%) at pH 7, and incubated at 25°C for five days. For marine bacteria, 2 µL of each bacterial suspension (OD_620_ = 0.08) was inoculated onto three sterile filter paper discs (6 mm), which were then placed on Petri dishes containing NBRIP medium supplemented with Ca_3_PO_4_ and NaCl (2% and 5%) at pH 7, and incubated at 25°C for three days. As negative controls, filter paper discs containing NaCl (0.8%) were used for bacteria, and PDA agar discs were used for fungi. Phosphate-solubilizing activity was expressed as the phosphate solubilization index (PSI), defined as PSI = A/B, where A represents the total diameter of the halo (phosphate solubilization zone) including the colony, and B corresponds to the colony diameter alone (Ulloa-Muñoz et al., 2020).

#### 2.3.7 Production of hydrolytic enzymes

##### 2.3.7.1 Cellulolytic activity

For marine fungi, three mycelial discs (5 mm) were placed on Petri dishes containing Potato Dextrose Agar (PDA) supplemented with 5% carboxymethyl cellulose (CMC) and incubated at 25°C for five days. For endophytic bacteria, 2 µL of each bacterial suspension (OD_620_ = 0.08) was inoculated onto three sterile filter paper discs (6 mm), which were then placed on Petri dishes containing Tryptic Soy Agar (TSA) supplemented with 5% carboxymethyl cellulose (CMC) and NaCl (2% and 5%), and incubated at 25 °C for three days (Ulloa-Muñoz et al., 2020). As negative controls, filter paper discs containing NaCl (0.8%) were used for bacteria, and PDA agar discs were used for fungi. Cellulolytic activity was assessed following the Congo Red staining method (Tamariz-Angeles, 2014). A clear halo surrounding the disc, excluding the diameter of bacterial growth, was considered a positive indication of cellulose degradation.

##### 2.3.7.2 Pectinolytic activity

This assay was performed according to Pal et al. (2022), with subtle modifications. For marine fungi, three mycelial discs (5 mm) were placed on Petri dishes containing Potato Dextrose Agar (PDA) supplemented with 0.3% citrus pectin and NaCl (2% and 5%), and incubated at 25 °C for five days. For endophytic bacteria, 2 µL of each bacterial suspension (OD_620_ = 0.08) was inoculated onto three sterile filter paper discs (6 mm), which were then placed on Petri dishes containing Tryptic Soy Agar (TSA) supplemented with 0.3% citrus pectin and NaCl (2% and 5%), and incubated at 25°C for three days. As negative controls, filter paper discs containing NaCl (0.8%) were used for bacteria, and PDA agar discs were used for fungi. The hydrolysis zone was visualized using the Congo Red method with 0.2% (w/v) Congo Red solution, followed by washing with 0.8% NaCl containing 1% (v/v) acetic acid (Kumar et al., 2022).

##### 2.3.7.3 Proteolytic activity

Proteolytic activity was evaluated following the methodology proposed by Ulloa-Muñoz et al. (2020). Petri dishes were prepared with milk agar medium composed of 0.5% casein peptone, 0.25% yeast extract, 0.1% dextrose, 8% liquid skim milk, and 1.5% agar-agar, supplemented with NaCl (2% and 5%). For marine fungi, three mycelial discs (5 mm) were placed on milk agar plates and incubated at 25°C for five days. For endophytic bacteria, 2 µL of each bacterial suspension (OD_620_ = 0.08) was absorbed onto three sterile filter paper discs, which were then placed on milk agar and incubated at 25°C for 48 hours. As negative controls, filter paper discs containing NaCl (0.8%) were used for bacteria, and PDA agar discs were used for fungi. The presence of a clear halo surrounding the disc indicated positive proteolytic activity.

##### 2.3.7.4 Amylolytic activity

This assay was carried out following the methodology described by Verma et al. (2017). Petri dishes containing starch agar medium (0.1% glucose, 0.5% peptone, 1.5% starch, and 1.5% agar-agar) were prepared and supplemented with NaCl (2% and 5%). For marine fungi, three mycelial discs (5 mm) were placed on starch agar plates and incubated at 25°C for five days. For endophytic bacteria, 2 µL of each bacterial suspension (OD_620_ = 0.08) was absorbed onto three sterile filter paper discs, which were then placed on starch agar plates and incubated at 25°C for three days. As negative controls, filter paper discs containing NaCl (0.8%) were used for bacteria, and PDA agar discs were used for fungi. Amylolytic activity was revealed using 5% Lugol’s iodine solution (Pal et al., 2022). The formation of a clear halo surrounding the disc was considered a positive result.

### 2.4 Phylogenetic analysis of selected fungi

Eight marine fungi were selected based on antibacterial, anti-yeast, and antifungal activity tests; IAA production; siderophore production; phosphate solubilization; and hydrolytic enzyme production. These fungi were cultured on PDA agar for DNA extraction using the CTAB protocol. PCR amplification of the ITS fragment was performed with the ITS1/ITS4 primer pair. The quality of the amplicons was verified by electrophoresis on 1.2% agarose gel, following the method described by Tamariz-angeles et al. (2023) with minor changes. Sequencing was conducted using the ITS1 and ITS4 primers. The obtained sequences were edited and assembled with Chromas Lite and CAP3, respectively. Reference sequences were retrieved from GenBank using BlastN (https://blast.ncbi.nlm.nih.gov/). According to their taxonomic grouping, sequences were aligned using ClustalX, while phylogenetic analysis and tree construction were carried out in MEGA 12 employing the Neighbor-Joining method, the Kimura-2 parameter model, and 1,000 bootstrap replicates (Vega-portalatino et al., 2024).

### 2.5 Effect of marine bacteria and fungi on *Zea mays* under salt stress

Based on the prioritized indole-3-acetic acid (IAA) production assay and other tests siderophore production, phosphate solubilization, hydrolytic enzyme production and evaluating antimicrobial activity, three marine fungi (C21, L3, and L5) and two endophytic bacteria (*Bacillus* sp. YCFR5 and *Pantoea agglomerans* YAFL6) were selected for inoculation in maize (Zea mays) under salt stress conditions. For the marine fungi, cultures were streaked on PDA Petri plates and incubated at 25 °C for 5 days. Subsequently, spores were harvested and suspended in sterile NaCl solution (0.8%) to a final volume of 30 mL (OD_620_nm = 0.3) (Ulloa-Muñoz et al., 2020). Regarding the endophytic bacteria, each strain was inoculated into conical tubes containing 4 mL of TSB medium and incubated at 25 °C for 24 hours. The cultures were then centrifuged at 4500 × g for 5 minutes, and the resulting pellets were resuspended in sterile NaCl solution (0.8%) to a final volume of 30 mL (OD_620_nm = 0.5).

#### 2.5.1 Germination test

Thirty-five grams of Zea mays seeds were surface-sterilized with 0.1% mercuric chloride (HgCl_2_) for 3 minutes, followed by six successive washes with sterile distilled water for 5 minutes each. The disinfected seeds were then placed on sterile absorbent paper in Petri dishes. The seeds were soaked for 3 hours in 30 mL of the previously prepared marine fungal and endophytic bacterial suspensions. Subsequently, 20 maize seeds were transferred to Petri dishes containing water agar (1.5% agar-agar) supplemented with 1% NaCl and incubated at 25 °C under dark conditions for 72 hours (Ulloa-Muñoz et al., 2020). Seeds soaked in sterile NaCl solution (0.8%) were used as the negative control. All treatments were performed in triplicate. After incubation, the germination percentage was evaluated.

#### 2.5.2 Zea mays growth

After 5 days, twenty germinated seedlings were transferred to pots containing sterile vermiculite and maintained under a 16-hour light photoperiod at ambient temperature (ranging from 25 to 30 °C) for 18 days, following the methodology by Ulloa-Muñoz et al. (2020). Three replicates were prepared per treatment (each pot was considered as one experimental block). Irrigation was performed every 48 hours with sterile distilled water supplemented with 1% NaCl. At the end of the experimental period, shoot and root lengths, as well as fresh and dry weights of shoots and roots, were evaluated.

### 2.6 Statistical analysis

The data obtained from antimicrobial activity, IAA production, phosphate solubilization, hydrolytic enzyme production, and maize growth under salt stress were analyzed using the mean ± standard deviation (SD), ANOVA, and Tukey’s test (α = 0.05), employing the Statistical Package (SPSS) version 23, except for the siderophore production assay.

## 3 Results and discussion

### 3.1 Macroalgae collection and isolation of endophytic and epiphytic fungus

The macroalgae identified as *Caulerpa* sp., *Ahnfeltiopsis* sp., *Ulva* sp., and *Chondracanthus chamissoi* were collected from Yacila and Los Cangrejos beaches. A total of ten marine fungi were isolated from the attachment discs and fronds of the macroalgae collected at both sites, of which one marine fungus originated from *Caulerpa* sp. (Los Cangrejos Beach) and nine from *Caulerpa* sp., *Ahnfeltiopsis* sp., *Ulva* sp., and *C. chamissoi* (Yacila Beach), as shown in **Table 2** and **Figure 1A**. In addition, two epiphytic fungi were isolated from *Caulerpa* sp. (Yacila Beach).

**Table 2:** Endophytic fungi isolated from different marine macroalgae from Los Cangrejos and Yacila beaches, Piura – Peru.

Collection site	Species	Algae parts	Codes	Number of isolated fungi
*Los Cangrejos Beach*	*Caulerpa* sp.	Fixing disc	CCDF	1
		Stipe	CCFE	0
		Leaf	CCFL	0
	*Ulva* sp.	Stipe	CUFE	0
		Leaf	CUFL	0
	*Ahnfeltiopsis* sp.	Stipe	CAFÉ	0
		Leaf	CAFL	0
	*Chondracantus chamisoi*	Main shaft	CCFM	0
		Branching	CCFR	0
*Yacila Beach*	*Caulerpa* sp.	Fixing disc	YCDF	0
		Stipe	YCFE	2
		Leaf	YCFL	0
	*Ulva* sp.	Stipe	YUFE	1
		Leaf	YUFL	1
	*Ahnfeltiopsis* sp.	Stipe	YAFE	1
		Leaf	YAFL	3
	*Chondracantus chamisoi*	Main shaft	YCFM	0
		Branching	YCFR	1

Regarding the physicochemical parameters, Yacila Beach showed the following values: pH 7.65, temperature (T) 22.59 °C, total dissolved solids (TDS) 171 mg/L, dissolved oxygen (DO) 171 mg/L, atmospheric pressure (PSI) 14.72, electrical conductivity (EC) 60.9, and turbidity (Tu) 0.5, which differed from those recorded at Los Cangrejos Beach, as shown in **Table 1**.

### 3.2 Plant growth–promoting assays

#### 3.2.1 Antibacterial, anti-*Candida*, and antifungal assays

**Figure 2:**
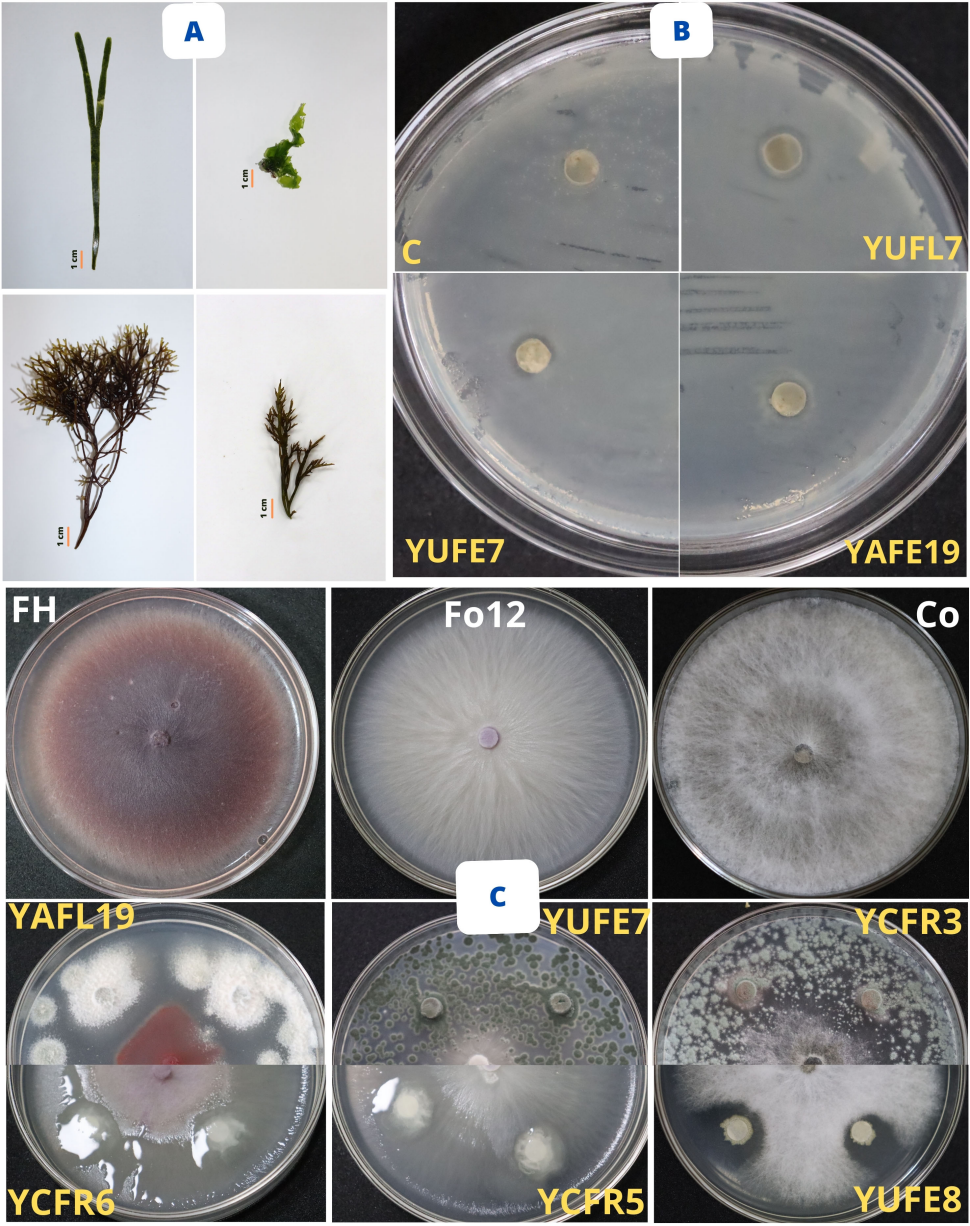
Marine macroalgae collected and antimicrobial potential of marine bacteria and fungi. **(A)** Marine macroalgae collected (*Caulerpa* sp., *Ahnfeltiopsis* sp., *Ulva* sp., and *Chondracanthus chamissoi*) from Yacila and Los Cangrejos beaches, Piura, Peru. **(B)** Antibacterial potential of marine fungi. Control: *Staphylococcus aureus* ATCC25923; YUFL7 inhibiting *S. aureus* ATCC700699; YUFE7 inhibiting *Staphylococcus epidermidis* ATCC12228; YAFE19 inhibiting *Escherichia coli* O157:H7. **(C)** Antifungal activity of marine microbes. FH, *Fusarium* sp. H inhibited by fungus YAFL19 and bacterium *Bacillus* sp. YCFR6; Fo12: *Fusarium oxysporum* CTLM12 inhibited by fungus YUFE7 and bacterium *Bacillus* sp. YCFR5; Co: *Colletotrichum* sp. inhibited by fungus YCFR3 and bacterium *Bacillus* sp. YUFE8.

Of the twelve marine fungal strains, YAFL13 and YUFL7 exhibited the highest inhibitory activity against *Staphylococcus aureus* ATCC700699, as did strains YAFL13 and YUFE7 against *S. aureus* ATCC25923. Likewise, YAFE19, YUFE7, and YUFL7 showed strong inhibition against *Staphylococcus epidermidis* ATCC12228. In addition, strains YAFE19, YUFE7, YUFL7, and YAFL19 demonstrated marked inhibitory effects against *Escherichia coli* O157:H7, followed by YUFL7, which exhibited strong inhibition against *Candida albicans* ATCC90028. However, no inhibitory activity was observed against *Enterococcus faecalis* ATCC29212, *S. aureus* ATCC29213, *Listeria monocytogenes* ATCC7644, *E. coli* ATCC10536, *Salmonella enterica* sv. Typhimurium ATCC14028, or *Candida tropicalis* ATCC750, as shown in **Table 3** and **Figure 2B**.

**Table 3:** Inhibitory activity of endophytic fungi against pathogenic bacteria and *Candida* species.

Organism	Strain	Sa99	Sa23	Se	EcH7	Ca28
Fungi	CCDF2	–	–	–	–	–
	YCFE3	–	–	–	–	–
	YCFE7	–	–	1.0 ± 0.0^c^	–	1.0 ± 0.0^b^
	YUFE7	2.3 ± 0.6^d^	2.0 ± 0.0^a,b^	3.3 ± 0.6^a^	2.0 ± 0.0^a^	–
	YUFL7	4.0 ± 0.0^a,b^	–	3.0 ± 0.0^a^	2.0 ± 0.0^a^	1.7 ± 0.6^a^
	YAFE19	3.3 ± 0.6^b,c^	1.7 ± 0.0^b,c^	3.3 ± 0.6^a^	2.3 ± 0.6^a^	–
	YAFL4	–	–	1.0 ± 0.0^c^	–	–
	YAFL13	4.7 ± 0.6^a^	2.7 ± 0.0^a^	2.0 ± 0.0^b^	–	–
	YAFL19	3.0 ± 0.0^c,d^	1.0 ± 0.0^c^	2.0 ± 0.0^b^	2.0 ± 0.0^a^	–
	YCFR3	–	–	2.7 ± 0.0^a,b^	–	–
	YFep2	–	–	–	–	–
	YFep3	–	–	–	–	–

Sa99, *Staphylococcus aureus* ATCC700699; Sa23, *Staphylococcus aureus* ATCC25923; Se, *Staphylococcus epidermidis* ATCC12228; EcH7, *Escherichia coli* O157:H7 and Ca28, *Candida albicans* ATCC90028. (–) no inhibitory activity. Values represent the mean of three replicates ± SD. Letters indicate groups with significant differences according to Tukey’s test (P < 0.05).

Regarding the marine fungi, strains YUFE7 and YAFE19 displayed strong inhibition against *Fusarium* sp. H. Likewise, YUFL7 showed high inhibitory activity against *Fusarium oxysporum* CTLM12, while YCFR3 presented strong inhibition against *Colletotrichum* sp. With respect to the endophytic bacteria, strains YUFE8, YCFR5, CUFE2, YCFR6, and YAFL6 inhibited *Fusarium* sp. H. Furthermore, YCFR showed strong inhibition against *F. oxysporum* CTLM12, as did strain YUFE8 against *Colletotrichum* sp., as shown in **Table 4** and **Figure 2C**.

**Table 4:** Inhibitory activity of endophytic microbes against filamentous fungi.

Organism	Strain	FH	Fo12	Co
Fungi	CCDF2	76.3 ± 1.4^d^	68.1 ± 1.3^d,e^	–
	YCFE3	61.3 ± 1.4^f^	60.6 ± 0.7^g^	–
	YCFE7	71.9 ± 0.7^e^	63.8 ± 1.4^f^	–
	YUFE7	83.8 ± 1.4^a^	89.4 ± 2.2^b^	–
	YUFL7	55.0 ± 0.0^g^	100.0 ± 0.0^a^	–
	YAFE19	81.3 ± 1.4^a,b^	69.4 ± 0.7^d^	–
	YAFL4	80.0 ± 0.0^b,c^	69.4 ± 0.7^d^	–
	YAFL13	2.5 ± 0.0^h^	68.1 ± 0.7^d,e^	–
	YAFL19	77.5 ± 2.0^c,d^	72.5 ± 0.0^c^	–
	YCFR3	2.5 ± 0.0^h^	73.8 ± 1.4^c^	71.3 ± 1.4^a^
	YFep2	79.4 ± 0.7^b,c^	66.3 ± 1.4^e,f^	–
	Yfep3	71.3 ± 1.4^e^	63.8 ± 1.4^f^	–
Bacteria	*Enterobacter* sp. YAFL1	59.0 ± 0.0^b^	65.4 ± 1.5^b^	–
	*Kluyvera ascorbata* YAFL9	–	–	–
	*Bacillus* sp. YCFE4	–	–	55.8 ± 0.7^b^
	*Bacillus* sp. YUFE8	64.7 ± 0.0^a^	–	64.1 ± 0.0^a^
	*Microbacterium paraoxydans* YCFE1	–	–	–
	*Bacillus* sp. YCFR5	65.4 ± 0.0^a^	68.6 ± 2.2^a^	–
	*Leclercia adecarboxylata* CUFE2	64.1 ± 0.0^a^	–	–
	*Bacillus* sp. YCFR6	62.8 ± 0.0^a,b^	64.1 ± 0.0^b,c^	–
	*Kluyvera ascorbata* YAFE21	–	–	–
	*Pantoea agglomerans* YAFL6	64.1 ± 3.0^a^	62.2 ± 0.7^c^	–

FH, *Fusarium* sp. H; Fo12, *F. oxysporum* CTLM12; Co, *Colletotricium* sp. (–) no inhibitory activity. Values represent the mean of three replicates ± SD. Letters indicate groups with significant differences according to Tukey’s test (P < 0.05).

#### 3.2.2 Production of IAA, siderophores, and phosphate solubilization

**Figure 3:**
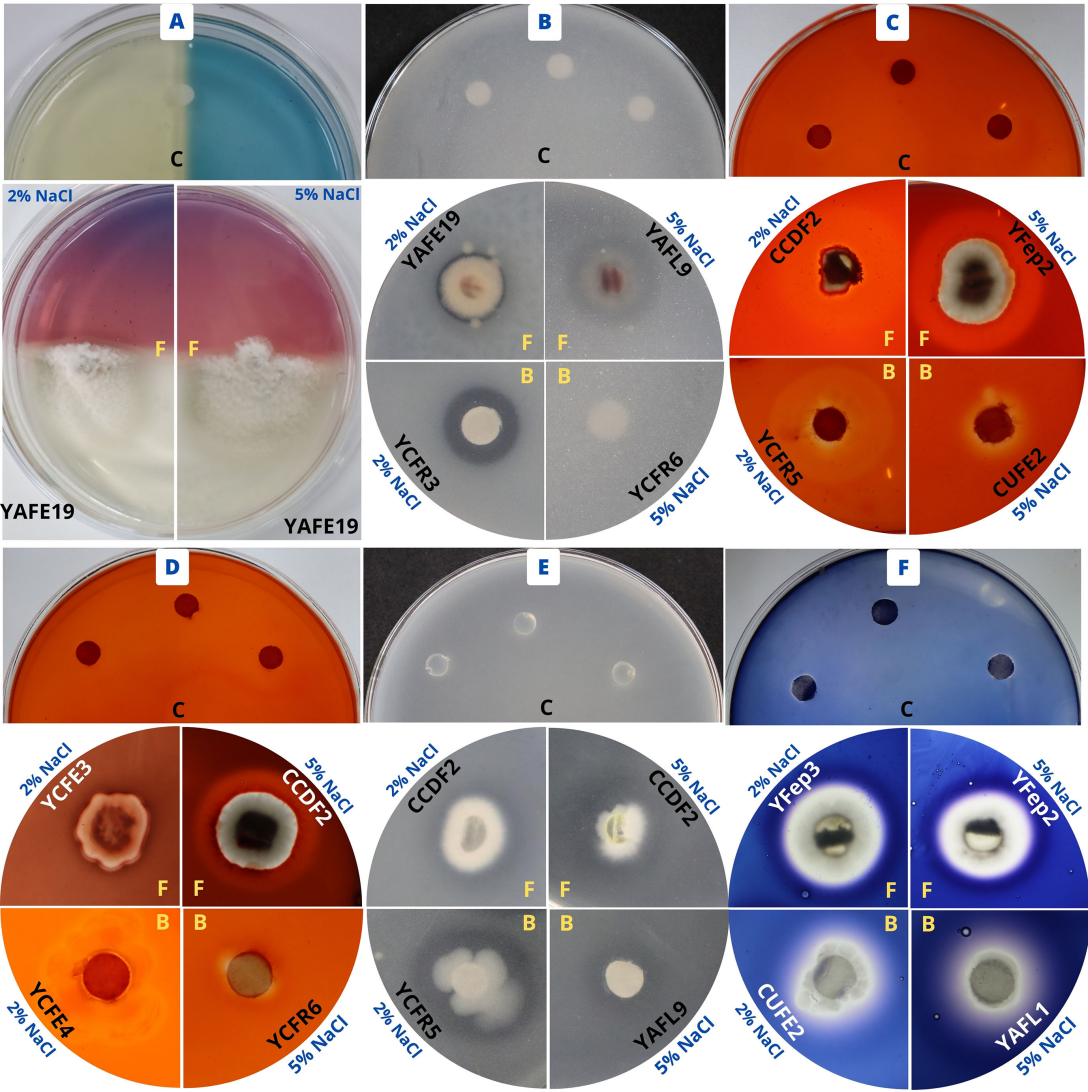
Plant growth-promoting assays of marine bacteria and fungi. **(A)** Siderophore production, **(B)** Phosphate solubilization, **(C)** Cellulolytic activity, **(D)** Pectinolytic activity, **(E)** Proteolytic activity, and **(F)** Amylolytic activity. C, Negative control; F, Fungi; and B, Bacteria.

The marine fungus YAFL13 exhibited the highest IAA synthesis (5.9 µg/mL) at 2% NaCl, while strains YCFE7 and YUFE7 showed strong siderophore production at 2% and 5% NaCl. Likewise, strains YCFR3 and YAFE19 achieved the highest phosphate solubilization (17 and 16.4 PSI, respectively) at 5% NaCl. Regarding the endophytic bacteria, YAFL6 demonstrated the highest IAA synthesis (22 µg/mL) at 2% NaCl, and strain YAFE21 showed the greatest phosphate solubilization (20 PSI) at 2% NaCl. However, no bacterial strain tested positive for siderophore production or phosphate solubilization at 5% NaCl, as shown in **Table 5** and **Figures 3A, B**.

**Table 5:** Production of IAA, siderophores, and phosphate solubilization by endophytic bacteria and fungi isolated from marine macroalgae.

Organism	Strain	IAA production (µg/ml)		Siderophore production		Phosphate solubilization (PSI)	
		2%	5%	2%	5%	2%	5%
Fungi	CCDF2	2.2 ± 0.3^b^	2.8 ± 0.0^b^	+	+	11.3 ± 0.1^b^	11.1 ± 0.1^c^
	YCFE3	–	–	++	+	–	–
	YCFE7	–	–	+++	+++	–	11.5 ± 0.1^c^
	YUFE7	–	–	+++	+++	11.4 ± 0.5^b^	11.3 ± 0.0^c^
	YUFL7	–	–	+++	++	11.3 ± 0.2^b^	11.3 ± 0.1^c^
	YAFE19	–	–	++	+++	11.7 ± 0.1^b^	16.4 ± 0.9^a^
	YAFL4	–	–	++	++	–	–
	YAFL13	5.9 ± 0.1^a^	4.6 ± 0.1^a^	+++	++	–	–
	YAFL19	–	–	++	++	13.0 ± 0.1^a^	13.0 ± 0.6^b^
	YCFR3	–	–	++	++	–	17.0 ± 1.3^a^
	YFep2	–	–	–	–	–	–
	Yfep3	–	–	–	–	–	–
Bacteria	*Enterobacter* sp. YAFL1	16.6 ± 0.4^b^	13.1 ± 0.1^b^	–	–	11.5 ± 0.6^d^	–
	*Kluyvera ascorbata* YAFL9	–	–	–	–	15.1 ± 1.1^b^	–
	*Bacillus* sp. YCFE4	16.8 ± 0.2^b^	7.7 ± 0.2^c^	–	–	12.2 ± 0.0^c,d^	–
	*Bacillus* sp. YUFE8	–	–	–	–	14.3 ± 0.0^b,c^	–
	*Microbacterium paraoxydans* YCFE1	–	–	–	–	–	–
	*Bacillus* sp. YCFR5	–	–	–	–	–	–
	*Leclercia adecarboxylata* CUFE2	–	–	–	–	15.1 ± 1.1^b^	–
	*Bacillus* sp. YCFR6	–	–	–	–	16.4 ± 1.2^b^	–
	*Kluyvera ascorbata* YAFE21	–	–	–	–	20.0 ± 0.0^a^	–
	*Pantoea agglomerans* YAFL6	22.0 ± 0.6^a^	13.5 ± 0.0^a^	–	–	15.0 ± 1.0^b^	–

NaCl concentration (2% and 5%). Production compared to the control: strong production (+++), moderate production (++), weak production (+), and no production (–). Values represent the mean of three replicates ± SD. Letters indicate groups with significant differences according to Tukey’s test (P < 0.05).

### 3.3 Synthesis of hydrolytic enzymes

Among the marine fungal strains, CCDF2 evidenced the highest degradation of cellulose (18 mm at 2% NaCl), pectin (6.3 mm at 5% NaCl), protein hydrolysis (8 mm at 5% NaCl), and starch (6 mm at 2% NaCl). Regarding the endophytic bacteria, strain YCFR5 demonstrated high cellulolytic activity (8.7 mm) at 2% NaCl, whereas YAFL6 displayed strong pectinolytic activity (4 mm), YCFR6 proteolytic activity (12.7 mm), and YAFL1 amylolytic activity (10 mm) at 5% NaCl, as shown in **Table 6** and **Figures 3C–F**.

**Table 6:** Production of hydrolytic enzymes by endophytic bacteria and fungi isolated from marine macroalgae.

Organism	Strain	Cellulolytic activity (mm)	Pectinolytic activity (mm)	Proteolytic activity (mm)	Amylolytic activity (mm)
Fungi	CCDF2	18.0 ± 0.0^a^ *(11.3 ± 0.6^b^)*	2.3 ± 0.6^a^ *(6.3 ± 0.6^a^)*	2.0 ± 0.0^a^ *(8.0 ± 0.0^a^)*	6.0 ± 0.0^a^ *(3.0 ± 0.0^a^)*
	YCFE3	–	–	–	–
	YCFE7	7.7 ± 0.6^d^ *(11.3 ± 0.6^b^)*	–	–	–
	YUFE7	–	–	*(4.7 ± 0.6^b,c,d^)*	–
	YUFL7	–	–	–	–
	YAFE19	*(9.3 ± 0.6^c^)*	–	*(5.7 ± 0.6^b^)*	–
	YAFL4	*(9.3 ± 0.6^c^)*	–	*(2.7 ± 0.6^e^)*	–
	YAFL13	9.3 ± 0.6^c^ *(10.7 ± 0.6^b,c^)*	–	2.0 ± 0.0^a^ *(5.3 ± 0.6^b,c^)*	–
	YAFL19	*(10.7 ± 0.6^b,c^)*	–	^-^	–
	YCFR3	*(15.3 ± 0.6^a^)*	–	–	–
	YFep2	3.0 ± 0.0^e^ *(12.0 ± 0.0^b^)*	2.7 ± 0.6^a^ *(6.0 ± 0.6^a^)*	2.3 ± 0.6^a^ *(4.0 ± 0.0^d^)*	3.0 ± 0.0^b^ *(3.3 ± 0.6^a^)*
	Yfep3	11.7 ± 0.6^b^ *(7.3 ± 0.6^d^)*	2.7 ± 0.6^a^ *(5.0 ± 0.6^b^)*	2.0 ± 0.0^a^ *(4.3 ± 0.6^c,d^)*	3.3 ± 0.0^b^ *(2.0 ± 0.0^b^)*
Bacteria	*Enterobacter* sp. YAFL1	*(4.0 ± 0.0^a,b^)*	–	–	3.7 ± 0.6^b^ *(10.0 ± 0.0^a^)*
	*Kluyvera ascorbata* YAFL9	–	–	*(5.3 ± 0.6^b^)*	*(2.0 ± 0.0^d^)*
	*Bacillus* sp. YCFE4	–	2.0 ± 0.6^NS^	–	–
	*Bacillus* sp. YUFE8	2.7 ± 0.6^c^ *(3.3 ± 0.6^b^)*	1.0 ± 0.0^NS^	–	–
	*Microbacterium paraoxydans* YCFE1	–	–	–	2.7 ± 0.6^c^ *(2.0 ± 0.0^d^)*
	*Bacillus* sp. YCFR5	8.7 ± 0.6^a^	–	5.7 ± 0.6^b^	6.7 ± 0.6^a^ *(1.0 ± 0.0^e^)*
	*Leclercia adecarboxylata* CUFE2	5.7 ± 0.6^b^ *(4.3 ± 0.6^a^)*	–	–	7.0 ± 0.0^a^ *(3.7 ± 0.6^c^)*
	*Bacillus* sp. YCFR6	–	*(2.7 ± 0.6^b^)*	8.3 ± 0.6^a^ *(12.7 ± 0.6^a^)*	–
	*Kluyvera ascorbata* YAFE21	*(1.0 ± 0.0^c^)*	–	*(4.3 ± 0.6^c^)*	1.0 ± 0.0^d^ *(1.7 ± 0.6^d,e^)*
	*Pantoea agglomerans* YAFL6	–	*(4.0 ± 0.0^a^)*	–	2.0 ± 0.0^c^ *(7.7 ± 0.6^b^)*

Values represent the mean of three replicates ± standard deviation (SD). Letters indicate groups with significant differences according to Tukey’s test (P < 0.05). NS: Not significant. Data in italics and parentheses correspond to 5% NaCl.

### 3.4 Molecular taxonomic identification of endophytic fungi

**Figure 4:**
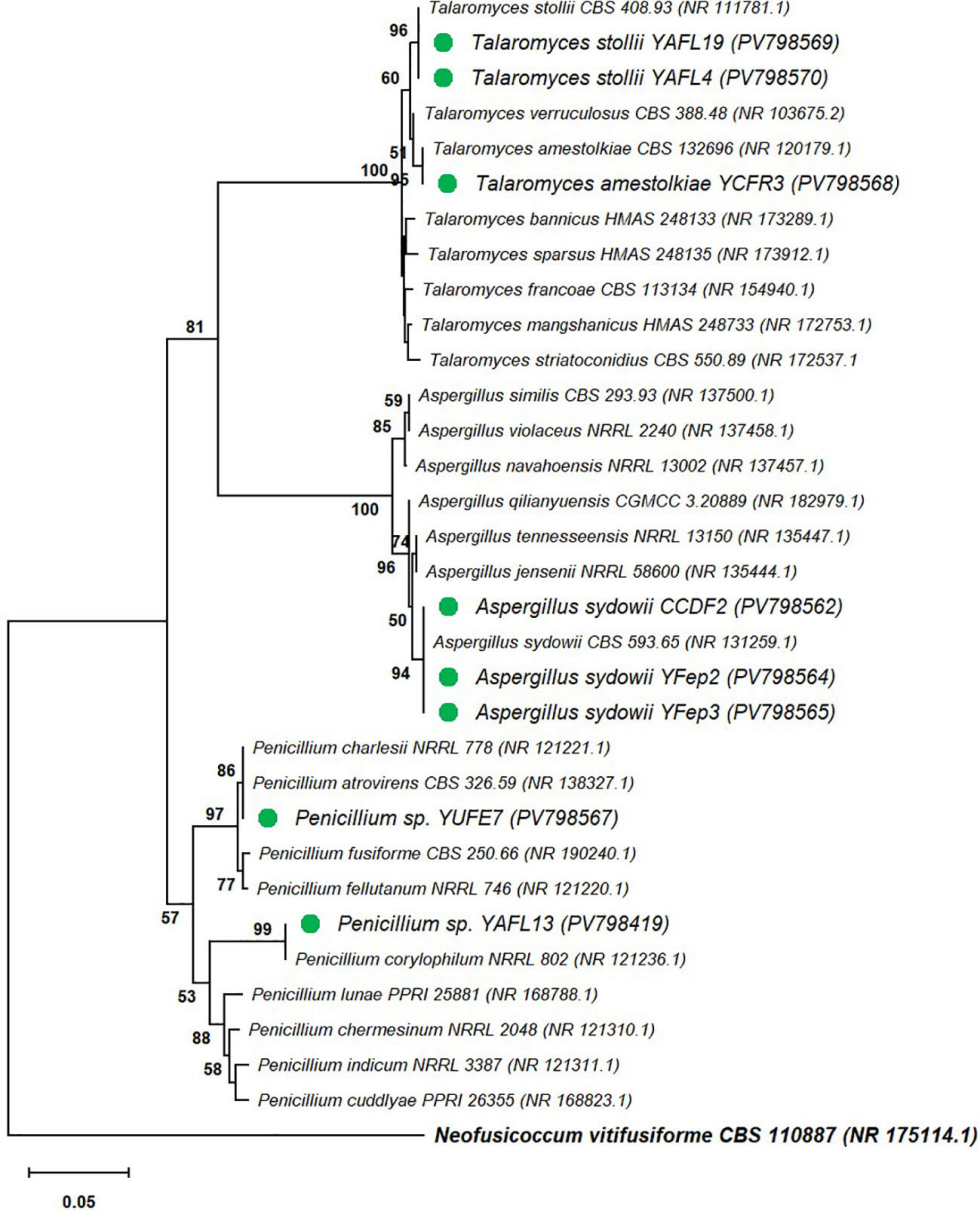
Phylogenetic analysis of marine fungi based on ITS sequences. Green dots represent the isolated strains, while the remaining sequences correspond to GenBank Type strains, with GenBank accession numbers shown in parentheses.

Of the eight marine fungi analyzed, two belonged to *Penicillium* sp. (YAFL13 and YUFE7), three to the genus *Talaromyces* (*T. stollii* YAFL19, YAFL4, and *T. amestolkiae* YCFR3), and three to *Aspergillus sydowii* (CCDF2, YFep2, and YFep3), as shown in **Figure 4**.

### 3.5 Effect of marine bacteria and fungi on the growth of Zea mays under salt stress

**Figure 5:**
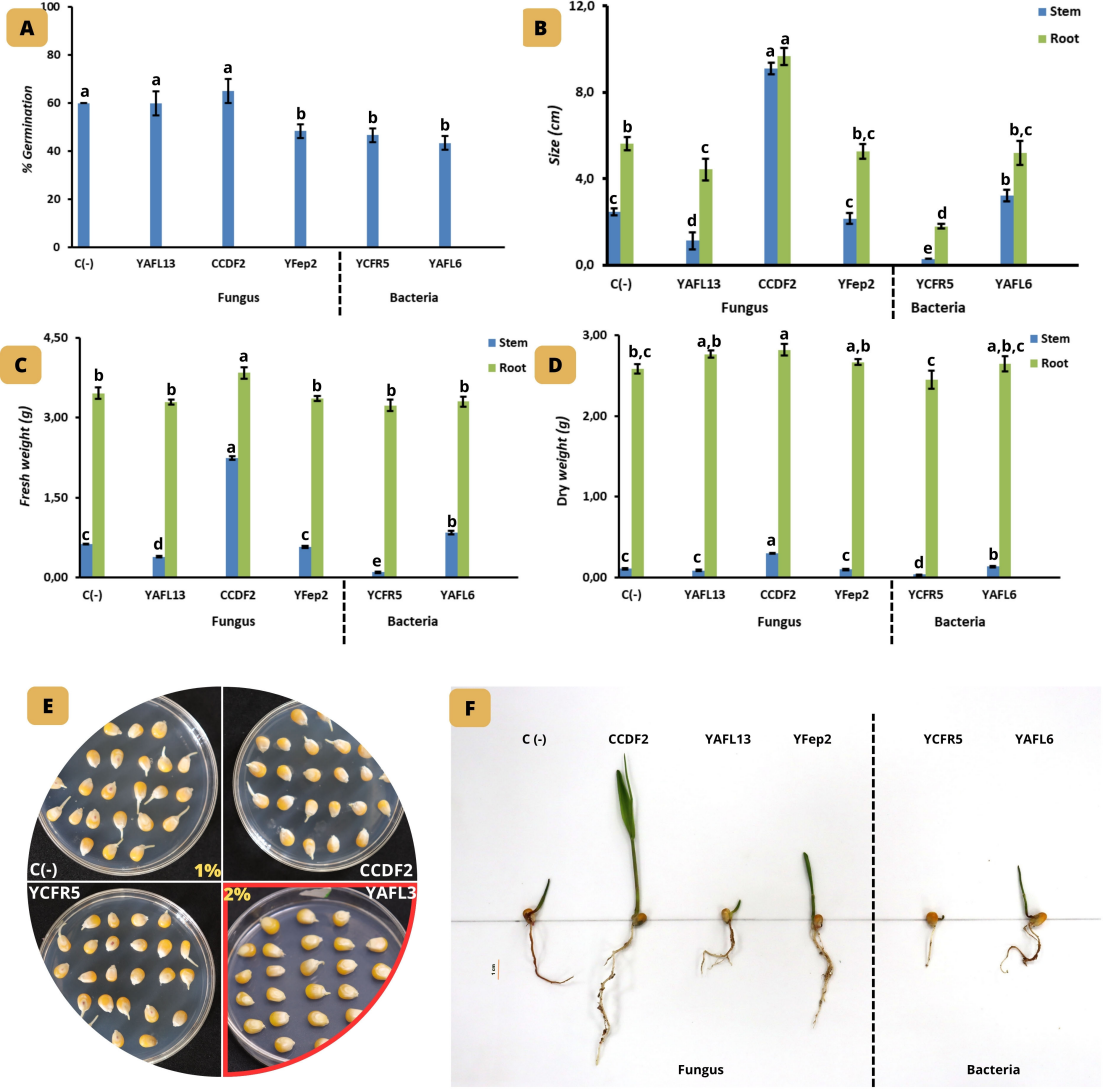
Effect of fungal and bacterial isolates from marine macroalgae on the growth of *Zea mays*. Germination percentage after 72 hours at 1% and 2% NaCl **(A–E),** stem and root length **(B)**, fresh and dry weight of stem and root **(C, D),** and seedling growth **(F)** after 18 days. YAFL13: *Penicillium* sp., CCDF2 and YFep2: *Aspergillus sydowii*, YCFR5: *Bacillus* sp., and YAFL6: *Pantoea agglomerans*. Values represent the mean of three replicates ± SD. Letters indicate groups with significant differences according to Tukey’s test (P < 0.05).

Among the three marine fungi and two endophytic bacteria evaluated, *Penicillium* sp. YAFL13 and *A. sydowii* CCDF2 displayed a germination percentage similar to the control (13%). However, regarding shoot and root length, *A. sydowii* CCDF2 reached 9.1 and 9.7 cm, respectively, compared to the control. In terms of fresh weight, *A. sydowii* CCDF2 showed the highest response in both shoot and root, with values of 2.24 and 3.84 g, respectively. For dry weight, *A. sydowii* CCDF2 also achieved the greatest response in the shoot (0.30 g), while *Penicillium* sp. YAFL13, *A. sydowii* CCDF2, *A. sydowii* YFep2, and the bacterium *P. agglomerans* YAFL6 recorded the highest root values, around 2.82 g, compared with the control plants subjected to 1% NaCl, as shown in **Figures 5A–F**.

## 4 Discussion

In recent years, research has focused on endophytic communities associated with marine algae to understand their structure, succession, and dynamics among bacteria, fungi, macroalgae, and the environment, although information on this topic remains limited (Abdelrazek et al., 2024). In this study, twelve fungal isolates were obtained from four macroalgae species (*Caulerpa* sp., *Ahnfeltiopsis* sp., *Ulva* sp., and *C. chamissoi*), most of which (75%) originated from the internal tissues of the stipe and blade of macroalgae collected at Yacila Beach. Vega-portalatino et al. (2024) a previous study reported that 71.7% of endophytic bacteria were isolated from the stipe of the same macroalgae collected at Yacila Beach, compared with those from Los Cangrejos Beach, showing consistency with the present findings. Several studies have reported the presence of endophytic fungi in marine macroalgae and their diversity depending on the organ and collection site (Gao and Zhang, 2023). For instance, Wong Chin et al. (2021) isolated 20 and 16 fungal strains from *Turbinaria conoides* and *Sargassum portieranum* collected from the beaches of Mauritius in the African country. Similarly, Harikrishnan et al. (2021) observed that endophytic fungi associated with the algae *Enteromorpha*, *Rhizoclonium*, and *Undaria* along the coast of Munambam, Kerala, India, belonged to *Basidiomycota*, while those from *Ulva* and *Chaetomorpha* collected at the Varkala coast, Kerala, India, belonged to *Ascomycota*—the latter being the most dominant group in marine mycology due to their ability to tolerate fluctuating saline conditions. The diversity of endophytic fungi may also be associated with the host genotype, tissue origin (Dastogeer et al., 2018), phenological stage (Wong Chin et al., 2021), geographical location (Wong Chin et al., 2021), nutrient availability, interaction with the host (Dastogeer et al., 2018), and anthropogenic factors.

Physicochemical parameters of water are standard indicators of water quality and play an essential role in maintaining the ecosystem’s balance with respect to its natural conditions, exerting a direct influence on fungal communities (Siriarchawatana et al., 2024). Regarding the physicochemical parameters at Yacila Beach, pH, T° and PSI and Ce were lower than those recorded at Los Cangrejos Beach, except for TDS, DO, and Tu. It is noteworthy that the highest number of fungal isolates was obtained from Yacila Beach. Wong Chin et al. (2021) reported the parameters for Mauritius Beach as temperature 25.4°C, pH 7.87, and salinity 3.47%, compared with Melvill Beach (temperature 24.5°C, pH 7.94, and salinity 3.5%), although no relationship was established between these factors and the number of endophytic isolates obtained from both sites. However, other authors have indicated that pH affects the redox potential of aquatic ecosystems and the productivity and biodiversity levels of algae (Abdelrazek et al., 2024), while some studies suggest that pH is not a significant factor influencing microbial communities (Scott F. et al., 2021). Higher temperature values (°C) can favor microbial growth in aquatic systems (Shahryari et al., 2024; Scott F. et al., 2021) depending on the optimal range for each organism. In contrast, there are no correlative reports linking PSI with microbial biodiversity. Nevertheless, high electrical conductivity can negatively affect enzymatic activity, leading to reduced microbial biomass (Al-Atrash et al., 2023). The elevated TDS content may be associated with increased concentrations of salts and organic and inorganic matter in the water (Ben et al., 2020). Similarly, high DO levels can enhance the proliferation rate of fungi (Fang et al., 2025). Turbidity is another factor that, when elevated, can protect microbes from inhibitory effects and stimulate their growth (Scott F. et al., 2021).

Microorganisms possess complex metabolic systems; therefore, their growth, activation of metabolic pathways, and rates of inhibitory metabolite production depend on their genetic constitution, physiology, available nutrients, and culture conditions (Tamariz-angeles et al., 2023). Regarding antibacterial activity, 25% of the marine fungi indicated strong inhibitory effects against *Staphylococcus aureus* ATCC700699, *S. aureus* ATCC25923, *S. epidermidis* ATCC12228, and *Escherichia coli* O157:H7. Concerning antifungal activity, 25% of the fungi and 16.6% of the marine bacteria inhibited *Fusarium* sp. H, *F. oxysporum* CTLM12, and *Colletotrichum* sp. through the dual culture assay (Nawrot-Chorabik et al., 2021). Likewise, five endophytic bacterial strains (YAFE21, YCFE4, FAYFL1, YAFL6, and CUFE2) isolated from *Caulerpa* sp., *Ahnfeltiopsis* sp., *Ulva* sp., and *C. chamissoi* evidenced inhibitory effects against both Gram-positive and Gram-negative pathogenic bacteria when tested using techniques that assess volatile/non-volatile compounds (OpT) and exclusively volatile compounds (VOCt) (Vega-portalatino et al., 2024), supporting the antimicrobial potential of these marine organisms. These findings are consistent with previous reports describing the inhibitory activity of marine endophytic fungi against *Bacillus cereus*, S. aureus, *Enterococcus faecalis*, *Salmonella typhimurium*, *Enterobacter cloacae*, *E. coli* (Wong Chin et al., 2021), *Pseudomonas aeruginosa*, *Cystobasidium minutum*, *Candida railenensis*, *Rigidoporus vinctus*, and *Grammothele fuligo*. This inhibitory action is possibly due to the production of antimicrobial compounds derived from genes horizontally transferred from their marine hosts (Harikrishnan et al., 2021). Such capacity could reduce infections caused by pathogenic bacteria (Chandrasekaran et al., 2023) by potentially disrupting cell membranes and walls, inhibiting and denaturing proteins and enzymes, damaging genetic material, altering actin filaments in the cytoskeleton, causing mitochondrial electrochemical depolarization, or interfering with biofilm formation (Vega-portalatino et al., 2023). These mechanisms collectively contribute to reduced pathogen colonization, activation of plant defense responses, and stimulation of plant growth, ultimately promoting soil and plant health (Chandrasekaran et al., 2023).

Among the plant growth-promoting tests, IAA (Yan-Yu et al., 2025) and siderophores (Park et al., 2023) are utilized by endophytic microbes to alleviate stress conditions by acting as communication signals, preparing cells to defend against imminent stressors (Yan-Yu et al., 2025). In this study, it was observed that 16.6% of marine fungi and 30% of marine bacteria produced IAA at 2% and 5% NaCl. Some studies have reported the IAA-producing ability in marine fungi such as *Agromyces mariniharenae* (Zhang et al., 2021), *Aspergillus flavus* (Vasanthkumar et al., 2024), *Fusarium proliferatum* (Nappo et al., 2025), and *Clonostachys rosea* (Liu et al., 2025a), and in bacteria such as *Shimia* sp., *Thalassobius* sp., *Phaeobacter* sp (Khalil et al., 2024), *Oceanobacillus oncorhynchi* W4 (Loganathan et al., 2024), *Agrobacterium*, and *Rhizobium* (Zhang et al., 2022). IAA is a plant hormone that can be biosynthesized from tryptophan via the intermediate indole-3-acetamide (IAM) (Nappo et al., 2025), being essential for regulating plant growth and cellular metabolism. It plays a vital role in cell division, cell elongation, photosynthetic regulation, and the induction of functional metabolism (Muhammad Aslam et al., 2022), promoting stem and root elongation toward nutrients (Khalil et al., 2024) under stress conditions (Yan-Yu et al., 2025). Regarding siderophore production, most fungi (83.3%) exhibited activity at 2% and 5% NaCl, whereas no marine bacteria tested positive for this trait. However, fungi such as *Pseudogymnoascus verrucosus* FAE27 (Montanares et al., 2025) and *Acremonium persicinum* F10 (Li and Li, 2021), as well as bacteria including *Bacillus taeanensis* SMI1, *Enterobacter* sp. AABM9 (Sarvepalli et al., 2023), *Marinobacter* sp. SVU_3 (Sarvepalli and Korrapati, 2023), and *Tenacibaculum maritimum* (Escribano et al., 2025), have been reported to produce siderophores. Siderophores are molecules that strongly bind iron, a limiting nutrient in marine environments (Park et al., 2023), typically present as hydroxamates (Chen et al., 2025). Knowledge regarding their use by marine microbes is limited (Park et al., 2023), but their production is known to promote plant growth by protecting against fungal infections and degrading pesticides, thereby benefiting roots in saline soils (Sarvepalli et al., 2023). Concerning phosphate solubilization, 58.3% of fungi showed activity at 2% and 5% NaCl, whereas bacteria (80%) responded only at 2% NaCl. Other studies have confirmed this capacity, where fungi such as *Cladosporium* sp. 1EM.P1, *Penicillium steckii* 5Y.P4 (de Souza Silva et al., 2025), and *Vishniacozyma victoriae* 2.L15 (da Silva et al., 2022), as well as bacteria including *Priestia megaterium* (H1), *Bacillus velezensis* (H2) (Mani et al., 2024), *Bacillus paramycoides*, (Li et al., 2023) and *Marinobacter* sp (Gonçalves et al., 2024), were able to solubilize phosphates, opening new possibilities for agriculture by reducing dependence on chemical fertilizers (de Souza Silva et al., 2025) and improving phosphorus availability in soil (Li et al., 2023) or water (Armandeh et al., 2022). All these assays demonstrated that halophilic marine strains could be employed as potential bioinoculants to enhance plant growth (Li et al., 2023; Mani et al., 2024) and mitigate salt stress within the scope of sustainable agriculture (Mani et al., 2024).

The production of hydrolytic enzymes by marine microbes under saline stress conditions has been shown to enhance the host’s induced systemic resistance (Khalil et al., 2021), solubilize insoluble nutrient forms to make them available to associated host plants (Koshila Ravi et al., 2022), and suppress diseases caused by soil-borne pathogens (Khalil et al., 2021). In this study, 75%, 25%, 50%, and 25% of fungi, as well as 50%, 40%, 40%, and 70% of marine bacteria, showed high cellulolytic, pectinolytic, proteolytic, and amylolytic activities at 2% and 5% NaCl. These results are supported by previous studies reporting that marine fungi such as *Penicillium* sp. MMS1906, *P. brevicompactum* MMS1910, *Penicillium* sp. MMS1986, *Aspergillus* sp. MMS1733, *Aspergillus* sp. MMS1785 (Le Strat et al., 2023), and *Trichoderma reesei* SL3-SP3.3 (Bahry et al., 2021) display high cellulolytic (Le Strat et al., 2023; Bahry et al., 2021), amylolytic, and proteolytic capacities (Bahry et al., 2021). Similarly, bacteria including *Marinagarivorans* sp. GE09 (Tsudome et al., 2023), *Aeromonas sobria* JC18 (Rohman et al., 2021), *Bacillus toyonensis* (Nursyirwani et al., 2021), *Vibrio navarensis*, and *Pseudomonas ganghwensis* (Fitriadi et al., 2023) degrade cellulose, pectin (Tsudome et al., 2023; Rohman et al., 2021), and proteins (Rohman et al., 2021; Nursyirwani et al., 2021; Fitriadi et al., 2023). The synthesis of cellulases by these organisms enables *in situ* decomposition of organic residues, improving soil fertility (Bautista-Cruz et al., 2024), or increases cellulose content in the intercellular matrix, enhancing cell wall structure and resistance to microbial degradation (Wu et al., 2023). Regarding pectinases, they can be applied for oil extraction, degumming, and biodescouring of cotton fiber (Singh et al., 2024), or to accelerate mucilage degradation, improving product consistency (Suo et al., 2025). Protease production by bacteria contributes to the lysis of pathogen fungal cell walls, acting as a biocontrol agent (Pathak et al., 2021), enhancing resistance to saline stress factors, delaying senescence, and improving productivity (Qi et al., 2025). Finally, amylase synthesis aids microbes in penetrating plant cells, improving their dispersal within the host (Khalil et al., 2021) and facilitating the hydrolysis of starch reserves into simple sugars, while also inhibiting the growth and development of pathogenic microbes (Khabirova et al., 2021).

The internal transcribed spacer (ITS) regions are widely used as universal primers for the identification of fungi and yeasts (Ezeonuegbu et al., 2022). In this study, eight fungi were selected for molecular taxonomic analysis, of which 25% belonged to the genus *Penicillium* sp. (YAFL13 and YUFE7), 25% to *Talaromyces stollii* (YAFL19, YAFL4), 12.5% to *T. amestolkiae* YCFR3, and 37.5% to *Aspergillus sydowii* (CCDF2, YFep2, and YFep3). *Penicillium* strains isolated from tobacco (Tarroum et al., 2022), wheat, sesame, and maize (Zhao et al., 2021) have demonstrated the ability to solubilize insoluble inorganic phosphorus, secrete siderophores and IAA, and produce hydrolytic enzymes (Zhao et al., 2021). Similarly, *Talaromyces* strains isolated from forest plants (e.g., *Mimosa himalayana*, *Desmodium triflorum*, *Mangifera indica*, *Geranium wallichianum*, *Crataeva nurvala*, *Terminalia arjuna*) (Kharkwal et al., 2024) or from maize rhizospheric soil (Sun et al., 2023) have shown IAA production, solubilization of nutrients such as phosphorus, zinc, and silica (Kharkwal et al., 2024), as well as phytase production (Sun et al., 2023). Although *A. sydowii* is a common fungus in marine ecosystems (Ibrahim et al., 2023), its ability to extract nitrogen and phosphorus from soil (Escobar Diaz et al., 2021), as well as inhibit the growth of *Rhizoctonia solani*, *R. nicotianae*, and *Pythium aphanidermatum* (Gharin et al., 2024), has been reported in studies associated with terrestrial biomes, similar to the the previously mentioned cases. Despite reports indicating that these marine fungi can promote plant growth, their role in protecting and enhancing the production of crops of agricultural importance remains largely unknown.

In this study, the strain *A. sydowii* CCDF2 displayed the highest performance in maize germination, stem and root length, and fresh and dry biomass at 1% NaCl. This result is supported by (Escobar Diaz et al., 2021), who demonstrated that the terrestrial fungus *A. sydowii* F112 enhanced phosphorus uptake in cotton plants, increasing total dry matter and respiratory activity. However, this may represent the first study reporting that this marine fungus is capable of increasing biomass in an agriculturally relevant crop under saline stress conditions.

## 5 Conclusions

The isolation of endophytic and epiphytic fungi in this study represents the first report from marine macroalgae such as *Ahnfeltiopsis* sp., *Ulva* sp., *C. chamissoi*, and *Caulerpa* sp. collected from Yacila and Los Cangrejos beaches, Piura, Peru. All isolated marine bacteria and fungi are reported for the first time as plant growth-promoting microorganisms, exhibiting high antimicrobial activity against *S. aureus* ATCC700699, *S. epidermidis* ATCC12228 and *F. oxysporum* CTLM12; synthesizing IAA; producing siderophores; solubilizing phosphates; and producing hydrolytic enzymes, particularly *Penicillium* sp. YAFL13, *A. sydowii* CCDF2, *A. sydowii* YFep2, *Bacillus* sp. YCFR5, and *Pantoea agglomerans* YAFL6. The maize bioassay suggests that the fungus *A. sydowii* CCDF2 has promising potential as a plant growth-promoting inoculant under saline stress conditions, enhancing plant protection. However, further functional genomic studies are required to elucidate the mechanisms underlying growth promotion in agronomically relevant crops.

## Data Availability

The datasets presented in this study can be found in online repositories. The names of the repository/repositories and accession number(s) can be found in the article/**Supplementary Material**.
