# Supplementary material for: Marine macroalgae-associated fungi from Yacila and Los Cangrejos beaches (Northern Peru) and previously selected marine bacteria evaluated as plant growth promoters in maize under saline stress
Source: Front Fungal Biol. 2026 Jan 14;6:1726850. doi: 10.3389/ffunb.2025.1726850 (PMC12847412; doi:10.3389/ffunb.2025.1726850)
Supplement: Supplementary file 1 [file DataSheet1.pdf]

Supplementary Material

1 Nucleotide sequences of the first group

>Penicillium_sp._YAFL13_(PV798419)

TTATTGTACCTTGTTGCTTCGGCGGGCCCGCCTCACGGCCGCCGGGGGGCTTCTGCCCTCTGGCCCGCGCCC
GCCGAAGACACCATTGAACACTGTCTGAAGATTGCAGTCTGAGCAATTAGCTAAATAAGTTAAAACTTTCA
ACAACGGATCTCTTGGTTCCGGCATCGATGAAGAACGCAGCGAAATGCGATACGTAATGTGAATTGCAGAA
TTCAGTGAATCATCGAGTCTTTGAACGCACATTGCGCCCCTTGGTATTCCGGGGGGCATGCCTGTCCGAGCG
TCATTGCTGCCCTCAAGCACGGCTTGTGTGTTGGGCCCCGTCCTCCTTCCCGGGGGACGGGCCCGAAAGGC
AGCGGCGGCACCGCGTCCGGTCCTCGAGCGTATGGGGCTTTGTCACCCGCTCTTGTAGGCCCGGCCGGCGC
TTGCCGACAACCATC

>Penicillium_corylophilum_NRRL_802_(NR_121236.1)

AAGGATCATTACCGAGTGAGGGCCCTCTGGGTCCAACCTCCCACCCATGTTTATTGTACCTTGTTGCTTC

GGCGGGCCCGCCTCACGGCCGCCGGGGGGCTTCTGCCCTCTGGCCCGCGCCCGCCGAAGACACCATTGAA

CACTGTCTGAAGATTGCAGTCTGAGCAATTAGCTAAATAAGTTAAAACTTTCAACAACGGATCTCTTGGT

TCCGGCATCGATGAAGAACGCAGCGAAATGCGATACGTAATGTGAATTGCAGAATTCAGTGAATCATCGA

GTCTTTGAACGCACATTGCGCCCCTTGGTATTCCGGGGGGCATGCCTGTCCGAGCGTCATTGCTGCCCTC

AAGCACGGCTTGTGTGTTGGGCCCCGTCCTCCTTCCCGGGGGACGGGCCCGAAAGGCAGCGGCGGCACCG

CGTCCGGTCCTCGAGCGTATGGGGCTTTGTCACCCGCTCTTGTAGGCCCGGCCGGCGCTTGCCGACAACC

ATCAATCTTTTTTCAGGTTGACCTCGGATCAGGTAGGGATACCCGCTGAACTTAAGCATATCAATAAGCG

GAGGAAAAGAAACCAACAGGGATTGCCTCAGTAACGGCGAGTGAA

>Penicillium_consobrinum_CBS_139144_(NR_144827.1)

AAGGATCATTACCGAGTGAGGGCCCTCTGGGTCCAACCTCCCACCCATGTTTATTGTACCTTGTTGCTTC

GGCGGGCCCGCCTCACGGCCGCCGGGGGGCTTCTGCCCTCTGGCCCGCGCCCGCCGAAGACACCATTGAA

CACTGTCTGAAGATTGCAGTCTGAGCAATTAGCTAAATAAGTTAAAACTTTCAACAACGGATCTCTTGGT

TCCGGCATCGATGAAGAACGCAGCGAAATGCGATACGTAATGTGAATTGCAGAATTCAGTGAATCATCGA

GTCTTTGAACGCACATTGCGCCCCTTGGTATTCCGGGGGGCATGCCTGTCCGAGCGTCATTGCTGCCCTC

AAGCACGGCTTGTGTGTTGGGCCCCGTCCTCCTTCCCGGGGGACGGGCCCGAAAGGCAGCGGCGGCACCG

CGTCCGGTCCTCGAGCGTATGGGGCTTTGTCACCCGCTCTTGTAGGCCCGGCCGGCGCTTGCCGACAACA

ATCAATCTTTTTTCAGGTTGACCTCGGATCAGGTAGGGATACCCGCTGAACTTAAGCATATCAATAA

>Penicillium_momoii_CBS_139157_(NR_144828.1)

AAGGATCATTACCGAGTGAGGGCCCTCTGGGTCCAACCTCCCACCCATGTTTATTGTACCTTGTTGCTTC

GGCGGGCCCGCCTCACGGCCGCCGGGGGGCTTCTGCCCTCTGGCCCGCGCCCGCCGAAGACACCATTGAA

CACTGTCTGAAGATTGCAGTCTGAGCAATTAGCTAAATAAGTTAAAACTTTCAACAACGGATCTCTTGGT

TCCGGCATCGATGAAGAACGCAGCGAAATGCGATACGTAATGTGAATTGCAGAATTCAGTGAATCATCGA

GTCTTTGAACGCACATTGCGCCCCTTGGTATTCCGGGGGGCATGCCTGTCCGAGCGTCATTGCTGCCCTC

AAGCACGGCTTGTGTGTTGGGCCCCGTCCTCCTTCCCGGGGGACGGGCCCGAAAGGCAGCGGCGGCACCG

CGTCCGGTCCTCGAGCGTATGGGGCTTTGTCACCCGCTCTTGTAGGCCCGGCCGGCGCTTGCCGACAACA

ATCAATCTTTTTTCAGGTTGACCTCGGATCAGGTAGGGATACCCGCTGAACTTAAGCAT

>Penicillium_rubefaciens_CBS_145.83_(NR_138310.1)

AAGGATCATTACCGAGTGAGGGCCCTCTGGGTCCAACCTCCCACCCATGTTTCTTGTACCTTGTTGCTTC

GGCGGGCCCGCCTCACGGCCGCCGGGGGGCTTCTGCCCTCTGGCCCGCGCCCGCCGAAGACACCATTGAA

CACTGTCTGAAGATTGCAGTCTGAGCAATTAGCTAAATAAGTTAAAACTTTCAACAACGGATCTCTTGGT

TCCGGCATCGATGAAGAACGCAGCGAAATGCGATACGTAATGTGAATTGCAGAATTCAGTGAATCATCGA

GTCTTTGAACGCACATTGCGCCCCTTGGTATTCCGGGGGGCATGCCTGTCCGAGCGTCATTGCTGCCCTC

AAGCACGGCTTGTGTGTTGGGCCCCGTCCTCCTTCCCGGGGGACGGGCCCGAAAGGCAGCGGCGGCACCG

CGTCCGGTCCTCGAGCGTATGGGGCTTTGTCACCCGCTCTTGTAGGCCCGGCCGGCGCTTGCCGACAACA

ATCAATCTTTTTTCAG

>Penicillium_xanthomelinii_CBS_139163_(NR_144831.1)

AAGGATCATTACCGAGTGAGGGCCCTCTGGGTCCAACCTCCCACCCATGTTTATCGTACCTTGTTGCTTC

GGCGGGCCCGCCTCACGGCCGCCGGGGGGCTTCTGCCCTCTGGCCCGCGCCCGCCGAAGACACCATTGAA

CACTGTCTGAAGATTGCAGTCTGAGCAATTAGCTAAATAAGTTAAAACTTTCAACAACGGATCTCTTGGT

TCCGGCATCGATGAAGAACGCAGCGAAATGCGATACGTAATGTGAATTGCAGAATTCAGTGAATCATCGA

GTCTTTGAACGCACATTGCGCCCCTTGGTATTCCGGGGGGCATGCCTGTCCGAGCGTCATTGCTGCCCTC

AAGCACGGCTTGTGTGTTGGGCCCCGTCCTCCTTCCCGGGGGACGGGCCCGAAAGGCAGCGGCGGCACCG

CGTCCGGTCCTCGAGCGTATGGGGCTTCGTCTTCCGCTCTTGTAGGCCCGGCCGGCGCTTGCCGACAACA

ATCAATCTTTTTTCAGGTTGACCTCGGATCA

>Penicillium_maclennaniae_CBS_198.81_(NR_138313.1)

AAGGATCATTACCGAGTGAGGGCCCTCTGGGTCCAACCTCCCACCCGTGTTTATCGTACCTTGTTGCTTC

GGCGGGCCCGCCTCACGGCCGCCGGGGGGCTTCTGCCCTCTGGCCCGCGCCCGCCGAAGACACCATTGAA

CGCTGTCTGAAGATTGCAGTCTGAGCAATTAGCTAAATAAGTTAAAACTTTCAACAACGGATCTCTTGGT

TCCGGCATCGATGAAGAACGCAGCGAAATGCGATACGTAATGTGAATTGCAGAATTCAGTGAATCATCGA

GTCTTTGAACGCACATTGCGCCCCTTGGTATTCCGGGGGGCATGCCTGTCCGAGCGTCATTGCTGCCCTC

AAGCACGGCTTGTGTGTTGGGCCCCGTCCTCCTTCCCGGGGGACGGGCCCGAAAGGCAGCGGCGGCACCG

CGTCCGGTCCTCGAGCGTATGGGGCTTCGTCTTCCGCTCTTGTAGGCCCGGCCGGCGCTTGCCGACAACA

ATCAATCTTTTTTCAG

>Penicillium_diabolicalicense_DAOMC_250542_(NR_171585.1)

TCCGTAGGTGAACCTGCGGAAGGATCATTACCGAGTGAGGGCCCTCTGGGTCCAACCTCCCACCCGTGTT

TATCGTACCTTGTTGCTTCGGCGGGCCCGCCTCACGGCCGCCGGGGGGCTTCTGCCCTCTGGCCCGCGCC

CGCCGAAGACACCATTGAACGCTGTCTGAAGATTGCAGTCTGAGCAATTAGCTAAATAAGTTAAAACTTT

CAACAACGGATCTCTTGGTTCCGGCATCGATGAAGAACGCAGCGAAATGCGATACGTAATGTGAATTGCA

GAATTCAGTGAATCATCGAGTCTTTGAACGCACATTGCGCCCCTTGGTATTCCGGGGGGCATGCCTGTCC

GAGCGTCATTGCTGCCCTCAAGCACGGCTTGTGTGTTGGGCCCCGTCCTCCTTCCCGGGGGACGGGCCCG

AAAGGCAGCGGCGGCACCGCGTCCGGTCCTCGAGCGTATGGGGCTTCGTCTTCCGCTCTTGTAGGCCCGG

CCGGCGCTTGCCGACAACAATCAATCTTTTTTCAGGTTGACCTCGGATCAGGTAGGGATACCCGCTGAAC

TTAAGCATATCAATAAGCGGAGGA

>Penicillium_burgense_CBS_325.89_(NR_171581.1)

AAGGATCATTACCGAGTGAGGGCCCTCTGGGTCCAACCTCCCACCCGTGTTTATCGTACCTTGTTGCTTC

GGCGGGCCCGCCTCACGGCCGCCGGGGGGCTTCTGCCCTCTGGCCCGCGCCCGCCGAAGACACCATTGAA

CGCTGTCTGAAGATTGCAGTCTGAGCAATTAGCTAAATAAGTTAAAACTTTCAACAACGGATCTCTTGGT

TCCGGCATCGATGAAGAACGCAGCGAAATGCGATACGTAATGTGAATTGCAGAATTCAGTGAATCATCGA

GTCTTTGAACGCACATTGCGCCCCTTGGTATTCCGGGGGGCATGCCTGTCCGAGCGTCATTGCTGCCCTC

AAGCACGGCTTGTGTGTTGGGCCCCGTCCTCCTTCCCGGGGGACGGGCCCGAAAGGCAGCGGCGGCACCG

CGTCCGGTCCTCGAGCGTATGGGGCTTCGTCTTCCGCTCTTGTAGGCCCGGCCGGCGCTTGCCGACAACA

ATCAATCTTTTTTTCAG

>Penicillium_raciborskii_NRRL_2150_(NR_121234.1)

AAGGATCATTACCGAGTGAGGGCCCTCTGGGTCCAACCTCCCACCCGTGTTTATCGTACCTTGTTGCTTC

GGCGGGCCCGCCTCACGGCCGCCGGGGGGCTTCTGCCCTCTGGCCCGCGCCCGCCGAAGACACCATTGAA

CGCTGTCTGAAGATTGCAGTCTGAGCAATTAGCTAAATAAGTTAAAACTTTCAACAACGGATCTCTTGGT

TCCGGCATCGATGAAGAACGCAGCGAAATGCGATACGTAATGTGAATTGCAGAATTCAGTGAATCATCGA

GTCTTTGAACGCACATTGCGCCCCTTGGTATTCCGGGGGGCATGCCTGTCCGAGCGTCATTGCTGCCCTC

AAGCACGGCTTGTGTGTTGGGCCCCGTCCTCCTTCCCGGGGGACGGGCCCGAAAGGCAGCGGCGGCACCG

CGTCCGGTCCTCGAGCGTATGGGGCTTCGTCTTCCGCTCTTGTAGGCCCGGCCGGCGCTTGCCGACAACA

ATCAATCTTTTTTTCAGGTTGACCTCGGATCAGGTAGGGATACCCGCTGAACTTAAGCATATCAATAAGC

GGAGGAAAAGAAACCAACAGGGATTGCCTCAGTAACGGCGAGTGAA

>Penicillium_velutinum_NRRL_2069_(NR_121235.1)

AAGGATCATTACCGAGTGAGGGCCCTCTGGGTCCAACCTCCCACCCGTGTTTATCGTACCTTGTTGCTTC

GGCGGGCCCGCCTCACGGCCGCCGGGGGGCTTCTGCCCTCTGGCCCGCGCCCGCCGAAGACACCATTGAA

CGCTGTCTGAAGATTGCAGTCTGAGCAATTAGCTAAATAAGTTAAAACTTTCAACAACGGATCTCTTGGT

TCCGGCATCGATGAAGAACGCAGCGAAATGCGATACGTAATGTGAATTGCAGAATTCAGTGAATCATCGA

GTCTTTGAACGCACATTGCGCCCCTTGGTATTCCGGGGGGCATGCCTGTCCGAGCGTCATTGCTGCCCTC

AAGCACGGCTTGTGTGTTGGGCCCCGTCCTCCTTCCCGGGGGACGGGCCCGAAAGGCAGCGGCGGCACCG

CGTCCGGTCCTCGAGCGTATGGGGCTTCGTCTTCCGCTCTTGTAGGCCCGGCCGGCGCTTGCCGACAACA

ATCAATCTTTTTTCAGGTTGACCTCGGATCAGGTAGGGATACCCGCTGAACTTAAGCATATCAATAAGCG

GAGGAAAAGAAACCAACAGGGATTGCCTCAGTAACGGCGAGTGAA

>Penicillium_terrenum_CBS_313.67_(NR_163675.1)

GGCTCAGGAGGGTTGGCAACGACCCCCCAGAGCCGGAAACTTGGTCAAACTCGGTCATTTAGAGGAAGTA

AAAGTCGTAACAAGGTTTCCGTAGGTGAACCTGCGGAAGGATCATTACCGAGTGAGGGCCCTCTGGGTCC

AACCTCCCACCCGTGTTTATCGTACCTTGTTGCTTCGGCGGGCCCGCCTCACGGCCGCCGGGGGGCTTCT

GCCCTCTGGCCCGCGCCCGCCGAAGACACCATTGAACGCTGTCTGAAGATTGCAGTCTGAGCAATTAGCT

AAATAAGTTAAAACTTTCAACAACGGATCTCTTGGTTCCGGCATCGATGAAGAACGCAGCGAAATGCGAT

ACGTAATGTGAATTGCAGAATTCAGTGAATCATCGAGTCTTTGAACGCACATTGCGCCCCTTGGTATTCC

GGGGGGCATGCCTGTCCGAGCGTCATTGCTGCCCTCAAGCACGGCTTGTGTGTTGGGCCCCGTCCTCCTT

CCCGGGGGACGGGCCCGAAAGGCAGCGGCGGCACCGCGTCCGGTCCTCGAGCGTATGGGGCTTCGTCTTC

CGCTCTTGTAGGCCCGGCCGGCGCTTGCCGACCACAATCAATCTTTTTTCAGGTTGACCTCGGATCAGGT

AGGGATACCCGCTGAACTTAAGCATATCAATAAGCGGAGGAAAAGAAACCAACAGGGATTGCCTCAGTAA

CGGCGAGTGAA

>Penicillium_melinii_FRR_2041_(NR_077155.1)

TCCGTAGGTGAACCTGCGGAAGGATCATTACCGAGTGAGGGCCCTCTGGGTCCAACCTCCCACCCGTGTT

TATCGTACCTTGTTGCTTCGGCGGGCCCGCCTCACGGCCGCCGGGGGGCTTCTGCCCTCTGGCCCGCGCC

CGCCGAAGACACCATTGAACGCTGTCTGAAGATTGCAGTCTGAGCAATTAGCTAAATAAGTTAAAACTTT

CAACAACGGATCTCTTGGTTCCGGCATCGATGAAGAACGCAGCGAAATGCGATACGTAATGTGAATTGCA

GAATTCAGTGAATCATCGAGTCTTTGAACGCACATTGCGCCCCTTGGTATTCCGGGGGGCATGCCTGTCC

GAGCGTCATTGCTGCCCTCAAGCACGGCTTGTGTGTTGGGCTCCGTCCTCCTTCCCGGGGGACGGGCCCG

AAAGGCAGCGGCGGCACCGCGTCCGGTCCTCGAGCGTATGGGGCTTCGTCTTCCGCTCTTGTAGGCCCGG

CCGGCGCTTGCCGACAACAATCAATCTTTTTCAGGTTGACCTCGGATCAGGTAGGGATACCCGCTGAAC

TTAAGCATATCAATAAGCGGAGGAAAAGAAACCAACAGGGATTGCCTC

>Penicillium_sp._YUFE7_(PV798567)

CGTGTATACTTACCGTGTTGCTTCGGCGGGCCCGCCTGTCAGGCCGCCGGGGGGCAACCGCCCCCGGGCCC
GCGCCCGCCGAAGACCCCAACGAACTCTTGTACCTTGCAGTCTGAGCGATAAGCATAAATTATTAAAACTT
TCAACAACGGATCTCTTGGTTCCGGCATCGATGAAGAACGCAGCGAAATGCGATAAGTAATGTGAATTGCA
GAATTCAGTGAATCATCGAGTCTTTGAACGCACATTGCGCCCCCTGGTATTCCGGGGGGCATGCCTGTCCG
AGCGTCATTACTGCCCTCAAGCCCGGCTTGTGTGTTGGGCGCCGCCCCCCCGGGGGCGGGCCCGAAAGGCA
GCGGCGGCACCGCGTCCGGTCCTCGAGCGTATGGGGCTTTGTCACCCGCCCGTAGGCCCGGCCGGCGCCCG
CCGAC

>Penicillium_charlesii_NRRL_778_(NR_121221.1)

AAGGATCATTACTGAGTGCGGGCCCTCTGGGTCCAACCTCCCACCCGTGTATACTTACCGTGTTGCTTCG

GCGGGCCCGCCTGTCAGGCCGCCGGGGGGCAACCGCCCCCGGGCCCGCGCCCGCCGAAGACCCCAACGAA

CTCTTGTACCTTGCAGTCTGAGCGATAAGCATAAATTATTAAAACTTTCAACAACGGATCTCTTGGTTCC

GGCATCGATGAAGAACGCAGCGAAATGCGATAAGTAATGTGAATTGCAGAATTCAGTGAATCATCGAGTC

TTTGAACGCACATTGCGCCCCCTGGTATTCCGGGGGGCATGCCTGTCCGAGCGTCATTACTGCCCTCAAG

CCCGGCTTGTGTGTTGGGCGCCGCCCCCCCGGGGGCGGGCCCGAAAGGCAGCGGCGGCACCGCGTCCGGT

CCTCGAGCGTATGGGGCTTTGTCACCCGCCCGTAGGCCCGGCCGGCGCCCGCCGACCCCCCAACCTTTTT

TTTTCAGGTTGACCTCGGATCAGGTAGGGATACCCGCTGAACTTAAGCATATCAATCAGCGGAGGAAAAG

AAACCAACAGGGATTGCCCTAGTAACGGCGAGTGAA

>Penicillium_atrovirens_CBS_326.59_(NR_138327.1)

AAGGATCATTACTGAGTGCGGGCCCTCTGGGTCCAACCTCCCACCCGTGTATACTTACCGTGTTGCTTCG

GCGGGCCCGCCTGTCAGGCCGCCGGGGGGCAACCGCCCCCGGGCCCGCGCCCGCCGAAGACCCCAACGAA

CTCTTGTACCTTGCAGTCTGAGCGATAAGCATAAATTATTAAAACTTTCAACAACGGATCTCTTGGTTCC
GGCATCGATGAAGAACGCAGCGAAATGCGATAAGTAATGTGAATTGCAGAATTCAGTGAATCATCGAGTC
TTTGAACGCACATTGCGCCCCCTGGTATTCCGGGGGGCATGCCTGTCCGAGCGTCATTACTGCCCTCAAG
CCCGGCTTGTGTGTTGGGCGCCGCCCCCCCGGGGGCGGGCCCGAAAGGCAGCGGCGGCACCGCGTCCGGT
CCTCGAGCGTATGGGGCTTTGTCACCCGCCCGTAGGCCCGGCCGGCGCCCGCCGACCCCCCAACCTTTTT
TTTTCAG

>Penicillium_fusiforme_CBS_250.66_(NR_190240.1)

AACGACCCCCCAGAGCCGGAAAGTTGTCCAAACTCGGTCATTTAGAGGAAGTAAAAGTCGTAACAAGGTT
TCCGTAGGTGAACCTGCGGAAGGATCATTACTGAGTGCGGGCCCTCTGGGTCCAACCTCCCACCCGTGTA
TACTTACCGTGTTGCTTCGGCGGGCCCGCCTGCCAGGCCGCCGGGGGGCAACCGCCCCCGGGCCCGCGCC
CGCCGAAGACCCCCACGAACTCTTGTACCTTGCAGTCTGAGCGATAAGCATAAATTATTAAAACTTTCAA
CAACGGATCTCTTGGTTCCGGCATCGATGAAGAACGCAGCGAAATGCGATAAGTAATGTGAATTGCAGAA
TTCAGTGAATCATCGAGTCTTTGAACGCACATTGCGCCCCCTGGTATTCCGGGGGGCATGCCTGTCCGAG
CGTCATTACTGCCCTCAAGCCCGGCTTGTGTGTTGGGCGCCGCCCCTCCGGGGGCGGGCCCGAAAGGCAG
CGGCGGCACCGCGTCCGGTCCTCGAGCGTATGGGGCTTTGTCACCCGCCCGTAGGCCCGGCCGGCGCCCG
CCGACCTCCTCCAACCTTTTTTTTTCAGGTTGACCTCGGATCAGGTAGGGATACCCGCTGAACTTAAGC
ATATCAATAAGCGGAGAAAAAGAAACCAACAGGGATTGCCCTAGTAACGGCGAGTGAA

>Penicillium_fellutanum_NRRL_746_(NR_121220.1)

AAGGATCATTACTGAGTGCGGGCCCTCTGGGTCCAACCTCCCACCCGTGTATACTTACCGTGTTGCTTCG
GCGGGCCCGCCTGCCAGGCCGCCGGGGGGCAACCGCCCCCGGGCCCGCGCCCGCCGAAGACCCCCACGAA
CTCTTTCTACCTTGCAGTCTGAGCGATAAGCATAAATTATTAAAACTTTCAACAACGGATCTCTTGGTTC
CGGCATCGATGAAGAACGCAGCGAAATGCGATAAGTAATGTGAATTGCAGAATTCAGTGAATCATCGAGT
CTTTGAACGCACATTGCGCCCCCTGGTATTCCGGGGGGCATGCCTGTCCGAGCGTCATTACTGCCCTCAA
GCCCGGCTTGTGTGTTGGGCGCCGCCCCCCCGGGGGCGGGCCCGAAAGGCAGCGGCGGCACCGCGTCCGG
TCCTCGAGCGTATGGGGCTTTGTCACCCGCCCGTAGGCCCGGCCGGCGCCCGCCGACCCCCTCCAACCTT
TTTTTTTTTCAGGTTGACCTCGGATCAGGTAGGGATACCCGCTGAACTTAAGCATATCAATAAGCGGAG
GAAAAGAAACCAACAGGGATTGCCCTAGTAACGGCGAGTGAA

>Penicillium_multicolor_CBS_501.73_(NR_111870.1)

AAGGATCATTACTGAGTGCGGGCCCTCTGGGTCCAACCTCCCACCCGTGTATACTTACCGTGTTGCTTCG

GCGGGCCCGCCTGCCAGGCCGCCGGGGGGCAACCGCCCCCGGGCCCGCGCCCGCCGAAGACCCCCACGAA

CTCTTTCTACCTTGCAGTCTGAGCGATAAGCATAAATTATTAAAACTTTCAACAACGGATCTCTTGGTTC

CGGCATCGATGAAGAACGCAGCGAAATGCGATAAGTAATGTGAATTGCAGAATTCAGTGAATCATCGAGT

CTTTGAACGCACATTGCGCCCCCTGGTATTCCGGGGGGCATGCCTGTCCGAGCGTCATTACTGCCCTCAA

GCCCGGCTTGTGTGTTGGGCGCCGCCCCCCCGGGGGCGGGCCCGAAAGGCAGCGGCGGCACCGCGTCCGG

TCCTCGAGCGTATGGGGCTTTGTCACCCGCCCGTAGGCCCGGCCGGCGCCCGCCGACCCCCTCCAACCTT

TTTTTTCAGGTTGACCTCGGATCAGGTAGGGATACCCGCTGAACTTAAGCATATCAA

>Penicillium_lunae_PPRI_25881_(NR_168788.1)

TCAGTGAGGCCTTCGGACTGGCTCAGGAGGGTTGGCAACGACCCCCCAGAGCCGGAAAGTTGGTCAAACT

CGGTCATTTAGAGGAAGTAAAAGTCGTAACAAGGTTTCCGTAGGTGAACCTGCGGAAGGATCATTACTGA

GTGCGGGCCCTCTGGGTCCAACCTCCCACCCGTGTATACCGTACCTTGTTGCTTCGGCGGGCCCGCCATT

CTGGCCGCCGGGGGGCACCCGCCCCCGGGCCCGCGCCCGCCGAAGACACCATTGAACGCTGTCTGAAGAT

TGCAGTCTGAGCGATAAGCAAAAATTAGTTAAAACTTTCAACAACGGATCTCTTGGTTCCGGCATCGATG

AAGAACGCAGCGAAATGCGATAAGTAATGTGAATTGCAGAATTCAGTGAATCATCGAGTCTTTGAACGCA

CATTGCGCCCCCTGGTATTCCGGGGGGCATGCCTGTCCGAGCGTCATTGCTGCCCTCAAGCCCGGCTTGT

GTGTTGGGCGCCGTCCCCCCGGGGACGGGCCCGAAAGGCAGCGGCGGCACCGCGTCCGGTCCTCGAGCGT

ATGGGGCTTTGTCACCCGCTCTGCAGGCCCGGCCGGCGCCAGCCGACCCCATCAACCCTTTTTTTCAGGT

TGACCTCGGATCAGGTAGGGATACCCGCTGAACTTAAGCATATCAATAAGCGGAGGAAAAGAAACCAACA

GGGATTGCCCTAGTAACGGCGAGTGAA

>Penicillium_indicum_NRRL_3387_(NR_121311.1)

AAGGATCATTACTGAGTGCGGGCCCTCTGGGTCCAACCTCCCACCCGTGTATACCGTACCTTGTTGCTTC

GGCGGGCCCGCCAGCCTTGGCCGCCGGGGGGCACCCGCCCCCGGGCCCGCGCCCGCCGAAGACATCATCG

AACGCTGTCTGAAGATTGCAGTCTGAGCGATAAGCACAAATTAGTTAAAACTTTCAACAACGGATCTCTT

GGTTCCGGCATCGATGAAGAACGCAGCGAAATGCGATAAGTAATGTGAATTGCAGAATTCAGTGAATCAT

CGAGTCTTTGAACGCACATTGCGCCCCCTGGTATTCCGGGGGGCATGCCTGTCCGAGCGTCATTGCTGCC

CTCAAGCCCGGCTTGTGTGTTGGGCGCCGTCCCCCCGGGGACGGGCCCGAAAGGCAGCGGCGGCACCGCG

TCCGGTCCTCGAGCGTATGGGGCTCTGTCACCCGCTCTGCAGGCCCGGCCGGCGCCAGCCGACCCCATCA

ACCCTTCTTTTTTCAGGTTGACCTCGGATCAGGTAGGGATACCCGCTGAACTTAAGCATATCAATAAGCG

GAGGAAAAGAAACCAACAGGGATTGCCCTAGTAACGGCGAGTGAA

>Penicillium_chermesinum_NRRL_2048_(NR_121310.1)

AAGGATCATTACTGAGTGCGGGCCCTCTGGGTCCAACCTCCCACCCGTGTATACCGTACCTTGTTGCTTC

GGCGGGCCCGCCAGCCTGGCCGCCGGGGGGCACCTGCCCCCGGGCCCGCGCCCGCCGAAGACATCATTGA

ACGCTGTCTGAAGATTGCAGTCTGAGCGATACGCATAAATTAGTTAAAACTTTCAACAACGGATCTCTTG

GTTCCGGCATCGATGAAGAACGCAGCGAAATGCGATAAGTAATGTGAATTGCAGAATTCAGTGAATCATC

GAGTCTTTGAACGCACATTGCGCCCCCTGGTATTCCGGGGGGCATGCCTGTCCGAGCGTCATTGCTGCCC

TCAAGCCCGGCTTGTGTGTTGGGCGCCGTCCCCCCGGGGACGGGCCCGAAAGGCAGCGGCGGCACCGCGT

CCGGTCCTCGAGCGTATGGGGCTCTGTCACCCGCTCCGCAGGCCCGGCCGGCGCCAGCCGACCCCCTCAA

CCCCTTTTTTTCAGGTTGACCTCGGATCAGGTAGGGATACCCGCTGAACTTAACATATCAATAAGCGGAG

GAAAAGAAACCAACAGGGATTGCCCTAGTAACGGCGAGTGAAG

>Penicillium_cuddlyae_PPRI_26355_(NR_168823.1)

TTCCGTAGGTGAACCTGCGGAAGGATCATTACTGAGTGCGGGCCCTCTGGGTCCAACCTCCCACCCGTGT

ATACCGTACCTTGTTGCTTCGGCGGGCCCGCCAGTCTGGCCGCCGGGGGGCACCTGCCCCCGGGCCCGCG

CCCGCCGGAGACATCATTGAACGCTGTCTGAAGATTGCAGTCTGAGCGATAAGCACAAATTAGTTAAAAC

TTTCAACAACGGATCTCTTGGTTCCGGCATCGATGAAGAACGCAGCGAAATGCGATAAGTAATGTGAATT

GCAGAATTCAGTGAATCATCGAGTCTTTGAACGCACATTGCGCCCCCTGGTATTCCGGGGGGCATGCCTG

TCCGAGCGTCATTGCTGCCCTCAAGCCCGGCTTGTGTGTTGGGCGCCGTCCCCCCGGGGACGGGCCCGAA

AGGCAGCGGCGGCACCGCGTCCGGTCCTCGAGCGTATGGGGCTCTGTCACCCGCTCTGCAGGCCCGGCCG

GCGCCAGCCGACCCCCTCAACCCTTTTTTTTTCAGGTTGACCTCGGATCAGGTAGGGATACCCGCTGAA

CTTAAGCATATCAATAAGCGGAGGAAAAGAAACCAACAGGGATTGCCCTAGTAACGGCGAGTGAA

>Talaromyces_tumuli_NRRL_62151_(NR_165528.1)

ATTACCGAGTGCGGGCCCTCGCGGCCCAACCTCCCACCCTTGTCTCTATACACCTGTTGCTTTGGCGGGC

CCACCGGGGCCACCTGGTCGCCGGGGGACGCACGTCCCCGGGCCCGCGCCCGCCGAAGCGCTCTGTGAAC

CCTGATGAAGATGGGCTGTCTGAGTACTATGAAAATTGTCAAAACTTTCAACAATGGATCTCTTGGTTCC

GGCATCGATGAAGAACGCAGCGAAATGCGATAAGTAATGTGAATTGCAGAATTCCGTGAATCATCGAATC

TTTGAACGCACATTGCGCCCCCTGGCATTCCGGGGGGCATGCCTGTCCGAGCGTCATTTCTGCCCTCAAG

CACGGCTTGTGTGTTGGGTGTGGTCCCCCCGGGGACCTGCCCGAAAGGCAGCGGCGACGTCCGTCTGGTC

CTCGAGCGTATGGGGCTCTGTCACTCGCTCGGGAAGGACCTGCGGGGGTTGGTCACCACCATGTTTTTAC

CACGGTTGACCTCGGATCAGGTAGGAGTTACCCGCTGAACTTAA

2 Nucleotide sequences of the second group

>Talaromyces_stollii_YAFL19_(PV798569)

TCTATACACCTGTTGCTTTGGCGGGCCCACCGGGGCCACCTGGTCGCCGGGGGACGTTCGTCCCGGGCCCG
CGCCCGCCGAAGCGCTCTGTGAACCCTGATGAAGATGGGCTGTCTGAGTACTATGAAAATTGTCAAAACTT
TCAACAATGGATCTCTTGGTTCCGGCATCGATGAAGAACGCAGCGAAATGCGATAAGTAATGTGAATTGCA
GAATTCCGTGAATCATCGAATCTTTGAACGCACATTGCGCCCCCTGGCATTCCGGGGGGCATGCCTGTCCG
AGCGTCATTTCTGCCCTCAAGCACGGCTTGTGTGTTGGGTGCGGTCCCCCCGGGGGCCTGCCCGAAAGGCA
GCGGCGACGTCCGTCTGGTCCTCGAGCGTATGGGGCTTTGTCACTCGCTCGGGAAGGACTGGCGGGG

> Talaromyces_stollii_CBS_408.93_(NR_111781.1)

AAGGATCATTACCGAGTGCGGGCCCCTCGTGGCCCAACCTCCCACCCTTGTCTCTATACACCTGTTGCTT

TGGCGGGCCCACCGGGGCCACCTGGTCGCCGGGGGACGTTCGTCCCCGGGCCCGCGCCCGCCGAAGCGCT

CTGTGAACCCTGATGAAGATGGGCTGTCTGAGTACTATGAAAATTGTCAAAACTTTCAACAATGGATCTC

TTGGTTCCGGCATCGATGAAGAACGCAGCGAAATGCGATAAGTAATGTGAATTGCAGAATTCCGTGAATC

ATCGAATCTTTGAACGCACATTGCGCCCCCTGGCATTCCGGGGGGCATGCCTGTCCGAGCGTCATTTCTG

CCCTCAAGCACGGCTTGTGTGTTGGGTGCGGTCCCCCCGGGGGCCTGCCCGAAAGGCAGCGGCGACGTCC

GTCTGGTCCTCGAGCGTATGGGGCTTTGTCACTCGCTCGGGAAGGACTGGCGGGGGTTGGTCACCACCAC

AAAATTTTACCACGGTTGACCTCGGATCAGGTAGGAGTTACCCGCTGAACTTAAGCATATCAATAAGCGG

AGGAAAAGAAACCAACCGGG

>Talaromyces_amestolkiae_CBS_132696_(NR_120179.1)

AAGGATCATTACCGAGTGCGGGCCCTCGTGGCCCAACCTCCCACCCTTGTCTCTATACACCTGTTGCTTT

GGCGGGCCCACCGGGGCCACCTGGTCGCCGGGGGACATCTGTCCCCGGGCCCGCGCCCGCCGAAGCGCTC

TGTGAACCCTGATGAAGATGGGCTGTCTGAGTACTATGAAAATTGTCAAAACTTTCAACAATGGATCTCT

TGGTTCCGGCATCGATGAAGAACGCAGCGAAATGCGATAAGTAATGTGAATTGCAGAATTCCGTGAATCA

TCGAATCTTTGAACGCACATTGCGCCCCCTGGCATTCCGGGGGGCATGCCTGTCCGAGCGTCATTTCTGC

CCTCAAGCACGGCTTGTGTGTTGGGTGCGGTCCCCCCGGGGACCTGCCCGAAAGGCAGCGGCGACGTCCG

TCTGGTCCTCGAGCGTATGGGGCTTTGTCACTCGCTCGGGAAGGACTGGCGGGGGTTGGTCACCACCAAA

ATTATACCACGGTTGACCTCGGATCAGGTAGGAGTTACCCGCTGAACTTAAGCATATCAATAAGCGGAGG

AAAAGAAACCAACCGGG

>Talaromyces_aspriconidius_CBS_141835_(NR_170774.1)

TTTCCGTAGGTGAACCTGCGGAAGGATCATTACCGAGTGCGGGCCCTCGCGGCCCAACCTCCCACCCTTG
TCTCTATACACCTGTTGCTTTGGCGGGCCCACCGGGGCCACCTGGTCGCCGGGGGACGTCGTCCCCGGGC
CCGCGCCCGCCGAAGCGCTCTGTGAACCCTGATGAAGATGGGCTGTCTGAGTACTATGAAAATTGTCAAA
ACTTTCAACAATGGATCTCTTGGTTCCGGCATCGATGAAGAACGCAGCGAAATGCGATAAGTAATGTGAA
TTGCAGAATTCCGTGAATCATCGAATCTTTGAACGCACATTGCGCCCCCTGGCATTCCGGGGGGCATGCC
TGTCCGAGCGTCATTTCTGCCCTCAAGCACGGCTTGTGTGTTGGGTGTGGTCCCCCCGGGGGCCTGCCCG
AAAGGCAGCGGCGACGTCCGTCTGGTCCTCGAGCGTATGGGGCTTTGTCACTCGCTCGGGAAGGACCTGC
GGGGGTTGGTCACCACCATATTTTTTTACCACGGTTGACCTCGGATCAGGTAGGAGTTACCCGCTGAAC
TTAA

>Talaromyces_australis_IBT_14256_(NR_147431.1)

AAGGATCATTACCGAGTGCGGGCCCTCGCGGCCCAACCTCCCACCCTTGTCTCTATACACCTGTTGCTTT
GGCGGGCCCACCGGGGCCACCTGGTCGCCGGGGGACGTCGTCCCCGGGCCCGCGCCCGCCGAAGCGCTCT
GTGAACCCTGATGAAGATGGGCTGTCTGAGTACTATGAAAATTGTCAAAACTTTCAACAATGGATCTCTT
GGTTCCGGCATCGATGAAGAACGCAGCGAAATGCGATAAGTAATGTGAATTGCAGAATTCCGTGAATCAT
CGAATCTTTGAACGCACATTGCGCCCCCTGGCATTCCGGGGGGCATGCCTGTCCGAGCGTCATTTCTGCC
CTCAAGCACGGCTTGTGTGTTGGGTGCGGTCCCCCCGGGGACCTGCCCGAAAGGCAGCGGCGACGTCCGT
CTGGTCCTCGAGCGTATGGGGCTCTGTCACTCGCTCGGGAAGGACCTGCGGGGGTTGGTCACCACCATAT
TTTACCACGGTTGACCTCGGATCAGGTAGGAGTTACCCGCTGAACTTAAGCATATCAATA

>Talaromyces_benedictus_MUM_23.44_(NR_199750.1)

AGAGGAAGTAAAAGTCGTAACAAGGTTTCCGTAGGTGAACCTGCGGAAGGATCATTACCGAGTGCGGGCC
CTCGTGGCCCAACCTCCCACCCTTGTCTCTATACACCTGTTGCTTTGGCGGGCCCACCGGGGCCACCTGG
TCGCCGGGGGACGTTCGTCCCCGGGCCCGCGCCCGCCGAAGCGCTCTGTGAACCCTGATGAAGATGGGCT
GTCTGAGTACTATGAAAATTGTCAAAACTTTCAACAATGGATCTCTTGGTTCCGGCATCGATGAAGAACG
CAGCGAAATGCGATAAGTAATGTGAATTGCAGAATTCCGTGAATCATCGAATCTTTGAACGCACATTGCG
CCCCCTGGCATTCCGGGGGGCATGCCTGTCCGAGCGTCATTTCTGCCCTCAAGCACGGCTTGTGTGTTGG
GTGTGGTCCCCCCCGGGGACCTGCCCGAAAGGCAGCGGCGACGTCCGTCTGGTCCTCGAGCGTATGGGGC
TTTGTCACTCGCTCGGGAAGGACCTGCGGAGGTTGGTCACCACCATATTTTACCACGGTTGACCTCGGAT
CAGGTAGGAGTTACCCGCTGAACTTAAGCATA

>Talaromyces_bannicus_HMAS_248133_(NR_173289.1)

TTTCCGTAGGTGAACCTGCGGAAGGATCATTACCGAGTGCGGGCCCTCGCGGCCCAACCTCCCACCCTTG

TCTCTATACACCTGTTGCTTTGGCGGGCCCACCGGGGCCACCCGGTCGCCGGGGGACGCTCGTCCCCGGG

CCCGCGCCCGCCGAAGCGCCCTGTGAACCCTGATGAAGATGGGCTGTCTGAGTACTATGAAAATTGTCAA

AACTTTCAACAATGGATCTCTTGGTTCCGGCATCGATGAAGAACGCAGCGAAATGCGATAAGTAATGTGA

ATTGCAGAATTCCGTGAATCATCGAATCTTTGAACGCACATTGCGCCCCCTGGCATTCCGGGGGGCATGC

CTGTCCGAGCGTCATTTCTGCCCTCAAGCACGGCTTGTGTGTTGGGTGCGGTCCCCCCGGGGACCTGCCC

GAAAGGCAGCGGCGACGTCCGTCTGGTCCTCGAGCGTATGGGGCTTTGTCACTCGCTCGGGAAGGACCTG

CGGGGGTTGGTCACCACCATATTTTACCACGGTTGACCTCGGATCAGGTAGGAGTTACCCGCTGAACTTA

A

>Talaromyces_mangshanicus_HMAS_248733_(NR_172753.1)

TTACCGAGTGCGGGCCCTCGCGGCCCAACCTCCCACCCTTGTCTATACACCTGTTGCTTTGGCGGGCCCA

CCGGGGCCACCTGGTCGCCGGGGGACGTCGTCCCCGGGCCCGCGCCCGCCGAAGCGCCCTGTGAACCCTG

ATGAAGATGGGCTGTCTGAGTACTATGAAAATTGTCAAAACTTTCAACAATGGATCTCTTGGTTCCGGCA

TCGATGAAGAACGCAGCGAAATGCGATAAGTAATGTGAATTGCAGAATTCCGTGAATCATCGAATCTTTG

AACGCACATTGCGCCCCCTGGCATTCCGGGGGGCATGCCTGTCCGAGCGTCATTTCTGCCCTCAAGCACG

GCTTGTGTGTTGGGTGTGGTCCCCCCGGGGACCTGCCCGAAAGGCAGCGGCGACGTCCGTCTGGTCCTCG

AGCGTATGGGGCTTTGTCACTCGCTCGGGAAGGACCTGCGGGGGTTGGTCACCACCATATTTTACCACGG

TTGACCTCGGATCAGGTAGGAGTTACCCGCTGAACTTAAGCATATCAATAA

>Talaromyces_pratensis_NRRL_62170_(NR_165529.1)

AAGGATCATTACCGAGTGCGGGCCCTCGCGGCCCAACCTCCCACCCTTGTCTCTATACACCTGTTGCTTT

GGCGGGCCCACCGGGGCCACCTGGTCGCCGGGGGACGCACGTCCCCGGGCCCGCGCCCGCCGAAGCGCTC

TGTGAACCCTGATGAAGATGGGCTGTCTGAGTACTATGAAAATTGTCAAAACTTTCAACAATGGATCTCT

TGGTTCCGGCATCGATGAAGAACGCAGCGAAATGCGATAAGTAATGTGAATTGCAGAATTCCGTGAATCA

TCGAATCTTTGAACGCACATTGCGCCCCCTGGCATTCCGGGGGGCATGCCTGTCCGAGCGTCATTTCTGC

CCTCAAGCACGGCTTGTGTGTTGGGTGTGGTCCCCCCGGGGACCTGCCCGAAAGGCAGCGGCGACGTCCG

TCTGGTCCTCGAGCGTATGGGGCTCTGTCACTCGCTCGGGAAGGACCTGCGGGGGTTGGTCACCACCACG

TTTTACCACGGTTGACCTCGGATCAGGTAGGAGTTACCCGCTGAACTTAA

>Talaromyces_tumuli_NRRL_62151_(NR_165528.1)

ATTACCGAGTGCGGGCCCTCGCGGCCCAACCTCCCACCCTTGTCTCTATACACCTGTTGCTTTGGCGGGC
CCACCGGGGCCACCTGGTCGCCGGGGGACGCACGTCCCCGGGCCCGCGCCCGCCGAAGCGCTCTGTGAAC
CCTGATGAAGATGGGCTGTCTGAGTACTATGAAAATTGTCAAAACTTTCAACAATGGATCTCTTGGTTCC
GGCATCGATGAAGAACGCAGCGAAATGCGATAAGTAATGTGAATTGCAGAATTCCGTGAATCATCGAATC
TTTGAACGCACATTGCGCCCCCTGGCATTCCGGGGGGCATGCCTGTCCGAGCGTCATTTCTGCCCTCAAG
CACGGCTTGTGTGTTGGGTGTGGTCCCCCCGGGGACCTGCCCGAAAGGCAGCGGCGACGTCCGTCTGGTC
CTCGAGCGTATGGGGCTCTGTCACTCGCTCGGGAAGGACCTGCGGGGGTTGGTCACCACCATGTTTTTAC
CACGGTTGACCTCGGATCAGGTAGGAGTTACCCGCTGAACTTAA

>Talaromyces_francoae_CBS_113134_(NR_154940.1)

AAGGATCATTACCGAGTGCGGGCCCTCGCGGCCCAACCTCCCACCCTTGTCTATACACCTGTTGCTTTGG
CGGGCCCACCGGGGCCACCCGGTCGCCGGGGGACATCGTCCCCGGGCCCGCGCCCGCCGAAGCGCTCTGT
GAACCCTGATGAAGATGGGCTGTCTGAGTACTATGAAAATTGTCAAAACTTTCAACAATGGATCTCTTGG
TTCCGGCATCGATGAAGAACGCAGCGAAATGCGATAAGTAATGTGAATTGCAGAATTCCGTGAATCATCG
AATCTTTGAACGCACATTGCGCCCCCTGGCATTCCGGGGGGCATGCCTGTCCGAGCGTCATTTCTGCCCT
CAAGCACGGCTTGTGTGTTGGGTGTGGTCCCCCCGGGGACCTGCCCGAAAGGCAGCGGCGACGTCCGTCT
GGTCCTCGAGCGTATGGGGCTTTGTCACTCGCTCGGGAAGGACCTGCGGGGGTTGGTCACCACCATATTT
TACCACGGTTGACCTCGGATCAGGTAGGAGTTACCCGCTGAACTTAAGCATATCAATAAGCGGAGGAAAA
GAAACCAACCGGG

>Talaromyces_sparsus_HMAS_248135_(NR_173912.1)

TTTCCGTAGGTGAACCTGCGGAAGGATCATTACCGAGTGCGGGCCCTCGTGGCCCAACCTCCCACCCTTG
TCTCTATACACCCGTTGCTTTGGCGGGCCCACCGGGGCCACCCGGTCGCCGGGGGACGTTCGTCCCCGGG
CCCGCGCCCGCCGAAGCGCTCTGTGAACCCTGATGAAGATGGGCTGTCTGAGTACCATGAAAATTGTCAA
AACTTTCAACAATGGATCTCTTGGTTCCGGCATCGATGAAGAACGCAGCGAAATGCGATAAGTAATGTGA
ATTGCAGAATTCCGTGAATCATCGAATCTTTGAACGCACATTGCGCCCCCTGGCATTCCGGGGGGCATGC
CTGTCCGAGCGTCATTTCTGCCCTCAAGCACGGCTTGTGTGTTGGGTGCGGTCCCCCCGGGGACCTGCCC
GAAAGGCAGCGGCGACGTCCGTCTGGTCCTCGAGCGTATGGGGCTTTGTCACTCGCTCGGGATGGACCTG
CGGGGGTTGGTCACCACCATATTTTACCACGGTTGACCTCGGATCAGGTAGGAGTTACCCGCTGAACTTA
A

>Talaromyces_striatoconidius_CBS_550.89_(NR_172537.1

GGCAAGTACACTGCATGGGGATTCCTACCGAGACCTCTCAGTAGAAACCTCACTGGTGAACGGTAACTGT

TCCGTAGGTGAACCTGCGGAAGGATCATTACCGAGTGCGGGCCCTCGTGGCCCAACCTCCCACCCTTGTC

TCTATACACCTGTTGCTTTGGCGGGCCCACCGGGGCCACCTGGTCGCCGGGGGACGAACGTCCCCGGGCC

CGCGCCCGCCGAAGCGCTCTGTGAACCCTGATGAAGATGGGCTGTCTGAGTACGATGAAAATTGTCAAAA

CTTTCAACAATGGATCTCTTGGTTCCGGCATCGATGAAGAACGCAGCGAAATGCGATAAGTAATGTGAAT

TGCAGAATTCCGTGAATCATCGAATCTTTGAACGCACATTGCGCCCCCTGGCATTCCGGGGGGCATGCCT

GTCCGAGCGTCATTTCTGCCCTCAAGCACGGCTTGTGTGTTGGGTGTGGTCCCCCCGGGGACCTGCCCGA

AAGGCAGCGGCGACGTCCGTCTGGTCCTCGAGCGTATGGGGCTTTGTCACTCGCTCGGGAAGGACCTGCG

GGGGTTGGTCACCACCATATTTTACCACGGTTGACCTCGGATCAGGTAGGAGTTACCCGCTGAACTTAAG

CATATCAATAAGCGGAGGAAAAGAAACCAACCGGGATTGCCTCAGTAACGGCGAGTGA

>Talaromyces_stollii_YAFL4_(PV798570)

GCGGAAGGATCATTACCGAGTGCGGGCCCCTCGTGGCCCAACCTCCCACCCTTGTCTCTATACACCTGTTGC
TTTGGCGGGCCCACCGGGGCCACCTGGTCGCCGGGGGACGTTCGTCCCCGGGCCCGCGCCCGCCGAAGCGC
TCTGTGAACCCTGATGAAGATGGGCTGTCTGAGTACTATGAAAATTGTCAAAACTTTCAACAATGGATCTCT
TGGTTCCGGCATCGATGAAGAACGCAGCGAAATGCGATAAGTAATGTGAATTGCAGAATTCCGTGAATCAT
CGAATCTTTGAACGCACATTGCGCCCCCTGGCATTCCGGGGGGCATGCCTGTCCGAGCGTCATTTCTGCCCT
CAAGCACGGCTTGTGTGTTGGGTGCGGTCCCCCCGGGGGCCTGCCCGAAAGGCAGCGGCGACGTCCGTCTG
GTCCTCGAGCGTATGGGGCTTTGTCACTCGCTCGGGAAGGACTGGCGGGGGTTGGTCACCACCACAAAATT
TTACCACGGTTGACCTCGGATCAGGTAGGAGTTACCCGCTGAACTTAAGCAT

>Talaromyces_amestolkiae_YCFR3_(PV798568)

GGCCCAACCTCCCACCCTTGTCTCTATACACCTGTTGCTTTGGCGGGCCCACCGGGGCCACCTGGTCGCCGG
GGGACATCTGTCCCCGGGCCCGCGCCCGCCGAAGCGCTCTGTGAACCCTGATGAAGATGGGCTGTCTGAGT
ACTATGAAAATTGTCAAAACTTTCAACAATGGATCTCTTGGTTCCGGCATCGATGAAGAACGCAGCGAAAT
GCGATAAGTAATGTGAATTGCAGAATTCCGTGAATCATCGAATCTTTGAACGCACATTGCGCCCCCTGGCA
TTCCGGGGGGCATGCCTGTCCGAGCGTCATTTCTGCCCTCAAGCACGGCTTGTGTGTTGGGTGCGGTCCCCC
CGGGGACCTGCCCGAAAGGCAGCGGCGACGTCCGTCTGGTCCTCGAGCGTATGGGGCTTTGTCACTCGCTC
GGGAAGGACTGGCGGGGGTTGGTCACCACCAAAATTATACCACGG

>Talaromyces_ruber_CBS_132704_(NR_111780.1)

AAGGATCATTACCGAGTGCGGGCCCTCGTGGCCCAACCTCCCACCCTTGTCTCTATACACCTGTTGCTTT

GGCGGGCCCACCGGGGTCACCTGGTCGCCGGGGGACAATCTGTCCCCGGGCCCGCGCCCGCCGAAGCGCT

CTGTGAACCCTGATGAAGATGGGCTGTCTGAGTACTATGAAAATTGTCAAAACTTTCAACAATGGATCTC

TTGGTTCCGGCATCGATGAAGAACGCAGCGAAATGCGATAAGTAATGTGAATTGCAGAATTCCGTGAATC

ATCGAATCTTTGAACGCACATTGCGCCCCCTGGCATTCCGGGGGGCATGCCTGTCCGAGCGTCATTTCTG

CCCTCAAGCACGGCTTGTGTGTTGGGTGCGGTCCCCCCGGGGACCTGCCCGAAAGGCAGCGGCGACGTCC

GTCTGGTCCTCGAGCGTATGGGGCTTTGTCACTCGCTCGGGAAGGGCTGGCGGGGGTTGGTCACCACCAA
AATTTTACCACGGTTGACCTCGGATCAGGTAGGAGTTACCCGCTGAACTTAAGCATATCAATAAGCGGAG
GAAAAGAAACCAACCGGG

>Talaromyces_verruculosus_CBS_388.48_(NR_103675.2)

TTCCGTAGGTGAACCTGCGGAAGGATCATTACCGAGTGCGGGCCCTCGGGCCCAACCTCCCACCCTTGTC
CTATACACCTGTTGCTTTGGCGGGCCCACCGGGGCCACCTGGTCGCCGGGGGACATCGTCCCCGGGCCTG
CGCCCGCCGAAGCGCTCTGTGAACCCTGATGAAGATGGGCTGTCTGAGTACTATGAAAATTGTCAAAACT
TTCAACAATGGATCTCTTGGTTCCGGCATCGATGAAGAACGCAGCGAAATGCGATAAGTAATGTGAATTG
CAGAATTCCGTGAATCATCGAATCTTTGAACGCACATTGCGCCCCCTGGCATTCCGGGGGGCATGCCTGT
CCGAGCGTCATTTCTGCCCTCAAGCACGGCTTGTGTGTTGGGTGCGGTCCCCCCGGGGACCTGCCCGAAA
GGCAGCGGCGACGTCCGTCGGTCCTCGAGCGTATGGGGCTTGTCACTCGCTCGGGAAGGACGGCGGGGGT
TGGTCACCACCAAAATTTACCACGGTTGACCTCGGATCAGGTAGGAGTTACCCGCTGAACTTAAGCATAT
CAATAAGCGGAGGAAAAGAAACCAACCGGGATTGCCTCAGTAACGGCGAGTGA

>Penicillium_cuddlyae_PPRI_26355_(NR_168823.1)

TTCCGTAGGTGAACCTGCGGAAGGATCATTACTGAGTGCGGGCCCTCTGGGTCCAACCTCCCACCCGTGT
ATACCGTACCTTGTTGCTTCGGCGGGCCCGCCAGTCTGGCCGCCGGGGGGCACCTGCCCCCGGGCCCGCG
CCCGCCGGAGACATCATTGAACGCTGTCTGAAGATTGCAGTCTGAGCGATAAGCACAAATTAGTTAAAAC
TTTCAACAACGGATCTCTTGGTTCCGGCATCGATGAAGAACGCAGCGAAATGCGATAAGTAATGTGAATT
GCAGAATTCAGTGAATCATCGAGTCTTTGAACGCACATTGCGCCCCCTGGTATTCCGGGGGGCATGCCTG
TCCGAGCGTCATTGCTGCCCTCAAGCCCGGCTTGTGTGTTGGGCGCCGTCCCCCCGGGGACGGGCCCGAA
AGGCAGCGGCGGCACCGCGTCCGGTCCTCGAGCGTATGGGGCTCTGTCACCCGCTCTGCAGGCCCGGCCG
GCGCCAGCCGACCCCCTCAACCCTTTTTTTTTCAGGTTGACCTCGGATCAGGTAGGGATACCCGCTGAA
CTTAAGCATATCAATAAGCGGAGGAAAAGAAACCAACAGGGATTGCCCTAGTAACGGCGAGTGAA

3 Nucleotide sequences of the third group

>Aspergillus_sydowii_CCDF2_(PV798562)

AACCTCCCACCCGTGAATACCTAACACTGTTGCTTCGGCGGGGAACCCCCTCGGGGGCGAGCCGCCGGGGA
CTACTGAACTTCATGCCTGAGAGTGATGCAGTCTGAGTCTGAATATAAAATCAGTCAAAACTTTCAACAAT
GGATCTCTTGGTTCCGGCATCGATGAAGAACGCAGCGAACTGCGATAAGTAATGTGAATTGCAGAATTCAG
TGAATCATCGAGTCTTTGAACGCACATTGCGCCCCCTGGCATTCCGGGGGGCATGCCTGTCCGAGCGTCATT
GCTGCCCATCAAGCCCGGCTTGTGTGTTGGGTCGTCGTCCCCCCCGGGGGACGGGCCCGAAAGGCAGCGGC

GGCACCGTGTCCGGTCCTCGAGCGTATGGGGCTTTGTCACCCGCTCGACTAGGGCCGGCCGGGCGCCAGCC GACGTCTCCA

>Aspergillus_sydowii_CBS_593.65_(NR_131259.1)

AGGATCATTACTGAGTGCGGGCTGCCTCCGGGCGCCCAACCTCCCACCCGTGAATACCTAACACTGTTGC

TTCGGCGGGGAACCCCCTCGGGGGCGAGCCGCCGGGGACTACTGAACTTCATGCCTGAGAGTGATGCAGT

CTGAGTCTGAATATAAAATCAGTCAAAACTTTCAACAATGGATCTCTTGGTTCCGGCATCGATGAAGAAC

GCAGCGAACTGCGATAAGTAATGTGAATTGCAGAATTCAGTGAATCATCGAGTCTTTGAACGCACATTGC

GCCCCCTGGCATTCCGGGGGGCATGCCTGTCCGAGCGTCATTGCTGCCCATCAAGCCCGGCTTGTGTGTT

GGGTCGTCGTCCCCCCCGGGGGACGGGCCCGAAAGGCAGCGGCGGCACCGTGTCCGGTCCTCGAGCGTAT

GGGGCTTTGTCACCCGCTCGACTAGGGCCGGCCGGGCGCCAGCCGACGTCTCCAACCATTTTTCTTCAGG

TTGACCTCGGATCAGGTAGG

>Aspergillus_tennesseensis_NRRL_13150_(NR_135447.1)

GCGGGCTGCCTCCGGGCGCCCAACCTCCCACCCGTGACTACCTAACACTGTTGCTTCGGCGGGGAGCCCT

CTCGGGGGCGAGCCGCCGGGGACTACTGAACTTCATGCCTGAGAGTGATGCAGTCTGAGTCTGAATATAA

AATCAGTCAAAACTTTCAACAATGGATCTCTTGGTTCCGGCATCGATGAAGAACGCAGCGAACTGCGATA

AGTAATGTGAATTGCAGAATTCAGTGAATCATCGAGTCTTTGAACGCACATTGCGCCCCCTGGCATTCCG

GGGGGCATGCCTGTCCGAGCGTCATTGCTGCCCATCAAGCCCGGCTTGTGTGTTGGGTCGTCGTCCCCCC

CGGGGGACGGGCCCGAAAGGCAGCGGCGGCACCGTGTCCGGTCCTCGAGCGTATGGGGCTTTGTCACCCG

CTCGATTAGGGCCGGCCGGGCGCCAGCCGACGTCTCCAACCATTTTCTTCAGGTTGACCTCGGATCAGGT

AGGGATACCCGCTGAACTTAAGCATATCAATAAGCGGAGGAAAAGAAACCAACCGGGATTGCCCCAGTAA

CGGCGAGTGAA

>Aspergillus_jensenii_NRRL_58600_(NR_135444.1)

AAGGATCATTACCGAGTGCGGGCTGCCTCCGGGCGCCCAACCTCCCACCCGTGACTACCTAACACTGTTG

CTTCGGCGGGGAGCCCTCTCGGGGGCGAGCCGCCGGGGACTACTGAACTTCATGCCTGAGAGTGATGCAG

TCTGAGTCTGAATATAAAATCAGTCAAAACTTTCAACAATGGATCTCTTGGTTCCGGCATCGATGAAGAA

CGCAGCGAACTGCGATAAGTAATGTGAATTGCAGAATTCAGTGAATCATCGAGTCTTTGAACGCACATTG

CGCCCCCTGGCATTCCGGGGGGCATGCCTGTCCGAGCGTCATTGCTGCCCATCAAGCCCGGCTTGTGTGT

TGGGTCGTCGTCCCCCCCGGGGGACGGGCCCGAAAGGCAGCGGCGGCACCGTGTCCGGTCCTCGAGCGTA

TGGGGCTTTGTCACCCGCTCGATTAGGGCCGGCCGGGCGCCAGCCGACGTCTCCAACCATTTTTCTTCAGG

TTGACCTCGGATCAGGTAGGGATACCCGCTGAACTTAAGCATATCAATAAGCGGAGGAAAAGAAACCAAC

CGGGATTGCCCCAGTAACGGCGAGTGAA

>Aspergillus_versicolor_ATCC_9577_(NR_131277.1)

TCCGTAGGTGAACCTGCGGAAGGATCATTACTGAGTGCGGGCTGCCTCCGGGCGCCCAACCTCCCACCCG

TGACTACCTAACACTGTTGCTTCGGCGGGGAGCCCTCTCGGGGGCGAGCCGCCGGGGACTACTGAACTTC

ATGCCTGAGAGTGATGCAGTCTGAGTCTGAATATAAAATCAGTCAAAACTTTCAACAATGGATCTCTTGG

TTCCGGCATCGATGAAGAACGCAGCGAACTGCGATAAGTAATGTGAATTGCAGAATTCAGTGAATCATCG

AGTCTTTGAACGCACATTGCGCCCCCTGGCATTCCGGGGGGCATGCCTGTCCGAGCGTCATTGCTGCCCA

TCAAGCCCGGCTTGTGTGTTGGGTCGTCGTCCCCCCCGGGGGACGGGCCCGAAAGGCAGCGGCGGCACCG

TGTCCGGTCCTCGAGCGTATGGGGCTTTGTCACCCGCTCGATTTAGGGCCGGCCGGGCGCCAGCCGACGT

CCAACCATTTTTCTTCAGGTTGACCTCGGATCAGGTAGGGATACCCGCTGAACTTAAGCATATCAATAAG

CGGAGGAAAAGAAACCAACCGGGATTGCCCC

>Aspergillus_tabacinus_NRRL _4791_(NR_135361.1)

AAGGATCATTACTGAGTGCGGGCTGCCTTCGGGCGCCCAACCTCCCACCCGTGACTACCTAACACTGTTG

CTTCGGCGGGGAGCCCTCTCGGGGGCGAGCCGCCGGGGACTACTGAACTTCATGCCTGAGAGTGATGCAG

TCTGAGTCTGAATATAAAATCAGTCAAAACTTTCAACAATGGATCTCTTGGTTCCGGCATCGATGAAGAA

CGCAGCGAACTGCGATAAGTAATGTGAATTGCAGAATTCAGTGAATCATCGAGTCTTTGAACGCACATTG

CGCCCCCTGGCATTCCGGGGGGCATGCCTGTCCGAGCGTCATTGCTGCCCATCAAGCCCGGCTTGTGTGT

TGGGTCGTCGTCCCCCCCGGGGGACGGGCCCGAAAGGCAGCGGCGGCACCGTGTCCGGTCCTCGAGCGTA

TGGGGCTTTGTCACCCGCTCGATTTAGGGCCGGCCGGGCGCCAGCCGACGTCCAACCATTTTTCTTCAGG

TTGACCTCGGATCAGGTAGGGATACCCGCTGAACTTAAGCATATCAATAAGCGGAGGAAAAGAAACCAAC

CGGGATTGCCCCAGTAACGGCGAGTGAA

>Aspergillus_griseoaurantiacus_CBS_138191_(NR_135459.1)

AAGGATCATTACTGAGTGCGGGCTGCCTTCGGGCGCCCAACCTCCCACCCGTGACTACCTAACACTGTTG

CTTCGGCGGGGAGCCCTCTCGGGGGCGAGCCGCCGGGGACTACTGAACTTCATGCCTGAGAGTGATGCAG

TCTGAGTCTGAATATAAAATCAGTCAAAACTTTCAACAATGGATCTCTTGGTTCCGGCATCGATGAAGAA

CGCAGCGAACTGCGATAAGTAATGTGAATTGCAGAATTCAGTGAATCATCGAGTCTTTGAACGCACATTG

CGCCCCCTGGCATTCCGGGGGGCATGCCTGTCCGAGCGTCATTGCTGCCCATCAAGCCCGGCTTGTGTGT

TGGGTCGTCGTCCCCCCCGGGGGACGGGCCCGAAAGGCAGCGGCGGCACCGTGTCCGGTCCTCGAGCGTA

TGGGGCTTTGTCACCCGCTCGATTTAGGGCCGGCCGGGCGCCAGCCGACGTCCAACCATTTTTCTTCAGG

TTGACCTCGGATCAGGTAGGGATA

>Aspergillus_protuberus_NRRL_3505_(NR_135353.1)

AAGGATCATTACTGAGTGCGGGCTGCCTCCGGGCGCCCAACCTCCCACCCGTGACTACCTAACACTGTTG

CTTCGGCGGGGAGCCCTCTCGGGGGCGAGCCGCCGGGGACTACTGAACTTCATGCCTGAGAGTGATGCAG

TCTGAGTCTGAATATAAAATCAGTCAAAACTTTCAACAATGGATCTCTTGGTTCCGGCATCGATGAAGAA

CGCAGCGAACTGCGATAAGTAATGTGAATTGCAGAATTCAGTGAATCATCGAGTCTTTGAACGCACATTG

CGCCCCCTGGCATTCCGGGGGGCATGCCTGTCCGAGCGTCATTGCTGCCCATCAAGCCCGGCTTGTGTGT

TGGGTCGTCGTCCCCCCCGGGGGACGGGCCCGAAAGGCAGCGGCGGCACCGTGTCCGGTCCTCGAGCGTA

TGGGGCTTTGTCACCCGCTCGATTTAGGGCCGGCCGGGCGCCAGCCGACGTCCAACCATTTTTCTTCAGG

TTGACCTCGGATCAGGTAGGGATACCCGCTGAACTTAAGCATATCAATAAGCGGAGGAAAAGAAACCAAC

CGGGATTGCCCCAGTAACGGCGAGTGAA

>Aspergillus_subversicolor_NRRL_58999_(NR_135446.1)

AAGGATCATTACCGAGTGCGGGCTGCCTTCGGGCGCCCAACCTCCCACCCGTGACTACCTAACACTGTTG

CTTCGGCGGGGAGCCCCCTTCCCGGGGGCGAGCCGCCGGGGACTACTGAACTTCATGCCTGAGAGTGATG

CAGTCTGAGTCTGAATATAAAATCAGTCAAAACTTTCAACAATGGATCTCTTGGTTCCGGCATCGATGAA

GAACGCAGCGAACTGCGATAAGTAATGTGAATTGCAGAATTCAGTGAATCATCGAGTCTTTGAACGCACA

TTGCGCCCCCTGGCATTCCGGGGGGCATGCCTGTCCGAGCGTCATTGCTGCCCATCAAGCCCGGCTTGTG

TGTTGGGTCGTCGTCCCCCCCCCGGGGGACGGGCCCGAAAGGCAGCGGCGGCACCGTGTCCGGTCCTCGA

GCGTATGGGGCTTTGTCACCCGCTCGATTAGGGCCGGCCGGGCGCCAGCCGACGTCTCCAACCATTTTTC

TTCAGGTTGACCTCGGATCAGGTAGGGATACCCGCTGAACTTAAGCATATCAATAAGCGGAGGAAAAGAA

ACCAACCGGGATTGCCCCAGTAACGGCGAGTGAA

>Aspergillus_recurvatus_NRRL_4902_(NR_135363.1)

AAGGATCATTACCGAGTGTGGGCTGCCTCCGGGCGCCCAACCTCCCACCCGTGACTACCTAACACTGTTG

CTTCGGCGGGGAGCCCCCCAGGGGGCGAGCCGCCGGGGACCACTGAACTTCATGCCTGAGAGTGATGCAG

TCTGAGTCTGAATACAAATCAGTCAAAACTTTCAACAATGGATCTCTTGGTTCCGGCATCGATGAAGAAC

GCAGCGAACTGCGATAAGTAATGTGAATTGCAGAATTCAGTGAATCATCGAGTCTTTGAACGCACATTGC

GCCCCCTGGCATTCCGGGGGGCATGCCTGTCCGAGCGTCATTGCTGCCCTTCAAGCCCGGCTTGTGTGTT

GGGTCGTCGTCCCCCCCGGGGGACGGGCCCGAAAGGCAGCGGCGGCACCGTGTCCGGTCCTCGAGCGTAT
GGGGCTTTGTCACCCGCTCGATTAGGGCCGGCCGGGCGCCAGCCGGCGTCTCCAACCTTATTTTTCTCAG
GTTGACCTCGGATCAGGTAGGGATACCCGCTGAACTTAAGCATATCAATAAGCGGAGGAAAAGAAACCAA
CCGGGATTGCCTCAGTAACGGCGAGTGAA

>Aspergillus_stercorarius_CBS_428.93_(NR_171593.1)

AACCTGCGGAAGGATCATTACCGAGTGTGGGCTGCCTCCGGGCGCCCAACCTCCCACCCGTGACTACCTA
ACACTGTTGCTTCGGCGGGGAGCCCCCCAGGGGGCGAGCCGCCGGGGACCACTGAACTTCATGCCTGAGA
GTGATGCAGTCTGAGTCTGAATACAAATCAGTCAAAACTTTCAACAATGGATCTCTTGGTTCCGGCATCG
ATGAAGAACGCAGCGAACTGCGATAAGTAATGTGAATTGCAGAATTCAGTGAATCATCGAGTCTTTGAAC
GCACATTGCGCCCCCTGGCATTCCGGGGGGCATGCCTGTCCGAGCGTCATTGCTGCCCTTCAAGCCCGGC
TTGTGTGTTGGGTCGTCGTCCCCCCCGGGGGACGGGCCCGAAAGGCAGCGGCGGCACCGTGTCCGGTCCT
CGAGCGTATGGGGCTTTGTCACCCGCTCGATTAGGGCCGGCCGGGCGCCAGCCGGCGTCTCCAACCTTAT
TTTTCTCAGGTTGACCTCGGATCAGGTAGGGATACCCGCTGAACTTAAGCATATCAATAAGCGGAGGA

>Aspergillus_falconensis_CBS_271.91_(NR_151790.1)

AACCTGCGGAAGGATCATTACCGAGTGCGGGCTGCCTCCGGGCGCCCAACCTCCCACCCGTGACTACCTA
ACACTGTTGCTTCGGCGGGGAGCCCCCCAGGGGGCGAGCCGCCGGGGACCACTGAACTTCATGCCTGAGA
GTGATGCAGTCTGAGTCTGAATACAAATCAGTCAAAACTTTCAACAATGGATCTCTTGGTTCCGGCATCG
ATGAAGAACGCAGCGAACTGCGATAAGTAATGTGAATTGCAGAATTCAGTGAATCATCGAGTCTTTGAAC
GCACATTGCGCCCCCTGGCATTCCGGGGGGCATGCCTGTCCGAGCGTCATTGCTGCCCTTCAAGCCCGGC
TTGTGTGTTGGGTCGTCGTCCCCCCCGGGGGACGGGCCCGAAAGGCAGCGGCGGCACCGTGTCCGGTCCT
CGAGCGTATGGGGCTTTGTCACCCGCTCGATTAGGGCCGGCCGGGCGCCAGCCGGCGTCTCCAACCTTAT
TTTTCTCAGGTTGACCTCGGATCAGGTAGGGATACCCGCTGAACTTAAGCATATCAATAAGCGGAGGA

>Aspergillus_dipodomyus_NRRL_66273_(NR_171615.1)

CATTACCGAGTGCGGGCTGCCTCCGGGCGCCCAACCTCCCACCCGTGACTACCTAACACTGTTGCTTCGG
CGGGGAGCCCCCCAGGGGGCGAGCCGCCGGGGACCACTGAACTTCATGCCTGAGAGTGATGCAGTCTGAG
TCTGAATACAAATCAGTCAAAACTTTCAACAATGGATCTCTTGGTTCCGGCATCGATGAAGAACGCAGCG
AACTGCGATAAGTAATGTGAATTGCAGAATTCAGTGAATCATCGAGTCTTTGAACGCACATTGCGCCCCC
TGGCATTCCGGGGGGCATGCCTGTCCGAGCGTCATTGCTGCCCTTCAAGCCCGGCTTGTGTGTTGGGTCG

TCGTCCCCCCCGGGGGACGGGCCCGAAAGGCAGCGGCGGCACCGTGTCCGGTCCTCGAGCGTATGGGGCT

TTGTCACCCGCTCGATTAGGGCCGGCCGGGCGCCAGCCGGCGTCTCCAACCTTATTTTTCTCAGGTTGAC

CTCGGATCAGGTAGGGATACCCGCTGAACTTAAGCATATC

>Aspergillus_qilianyuensis_CGMCC_3.20889_(NR_182979.1)

GATCATTACTGAGTGCGGGCTGCCTTCGGGCGCCCAACCTCCCACCCGTGACTACCACACACTGTTGCTT

CGGCGGGGAGCCCTCGGGCGAGCCGCCGGGGACTACTGAACTTCATGCCTGAGAGTGATGCAGTCTGAGT

CTGAATATAAAATCAGTCAAAACTTTCAACAATGGATCTCTTGGTTCCGGCATCGATGAAGAACGCAGCG

AACTGCGATAAGTAATGTGAATTGCAGAATTCAGTGAATCATCGAGTCTTTGAACGCACATTGCGCCCCC

TGGCATTCCGGGGGGCATGCCTGTCCGAGCGTCATTGCTGCCCATCAAGCCCGGCTTGTGTGTTGGGTCG

TCGTCCCCCCCGGGGGACGGGCCCGAAAGGCAGCGGCGGCACCGTGTCCGGTCCTCGAGCGTATGGGGCT

TTGTCACCCGCTCGATTTAGGGCCGGCCGGGCGCCAGCCGACGTCTCCAACCATTTTTCTTCAGGTTGAC

CTCGGATCAGGTAGGGATACCCGCTGAACTTAAGCATATCAA

>Aspergillus_similis_CBS_293.93_(NR_137500.1)

CGGAAGGATCATTACCGAGTGCGGGCTGCCTCCGGGCGCCCAACCTCCCACCCGTGACTACCTAACACTG

TTGCTTCGGCGGGGAGCCCCCCAGGGGGCGAGCCGCCGGGGACCACTGAACTTCATGCCTGAGAGTGATG

CAGTCTGAGTCTGAATACAAATCAGTCAAAACTTTCAACAATGGATCTCTTGGTTCCGGCATCGATGAAG

AACGCAGCGAACTGCGATAAGTAATGTGAATTGCAGAATTCAGTGAATCATCGAGTCTTTGAACGCACAT

TGCGCCCCCTGGCATTCCGGGGGGCATGCCTGTCCGAGCGTCATTGCTGCCCTTCAAGCCCGGCTTGTGT

GTTGGGTCGTCGTCCCCCTCGGGGGACGGGCCCGAAAGGCAGCGGCGGCACCGTGTCCGGTCCTCGAGCG

TATGGGGCTTTGTCACCCGCTCGATTAGGGCCGGCCGGGCGCCAGCCGGCGTCTCCAACCTT

>Aspergillus_violaceus_NRRL_2240_(NR_137458.1)

AAGGATCATTACCGAGTGCGGGCTGCCTCCGGGCGCCCAACCTCCCACCCGTGACTACCTAACACTGTTG

CTTCGGCGGGGAGCCCCCCAGGGGGCGAGCCGCCGGGGACCACTGAACTTCATGCCTGAGAGTGATGCAG

TCTGAGTCTGAATACAAATCAGTCAAAACTTTCAACAATGGATCTCTTGGTTCCGGCATCGATGAAGAAC

GCAGCGAACTGCGATAAGTAATGTGAATTGCAGAATTCAGTGAATCATCGAGTCTTTGAACGCACATTGC

GCCCCCTGGCATTCCGGGGGGCATGCCTGTCCGAGCGTCATTGCTGCCCTTCAAGCCCGGCTTGTGTGTT

GGGTCGTCGTCCCCCTCGGGGGACGGGCCCGAAAGGCAGCGGCGGCACCGTGTCCGGTCCTCGAGCGTAT

GGGGCTTTGTCACCCGCTCGATTAGGGCCGGCCGGGCGCCAGCCGGCGTCTCCAACCTTTTTTTCTCAGG

TTGACCTCGGATCAGGTAGGGATACCCGCTGAACTTAAGCATATCAATAAGCGGAGGAAAAGAAACCAAC

CGGGATTGCCTCAGTAACGGCGAGTGAA

>Aspergillus_navahoensis_NRRL_13002_(NR_137457.1)

AAGGATCATTACCGAGTGCGGGCTGCCTCCGGGCGCCCAACCTCCCACCCGTGACTACCTAACACTGTTG

CTTCGGCGGGGAGCCCCCCAGGGGGCGAGCCGCCGGGGACCACTGAACTTCATGCCTGAGAGTGATGCAG

TCTGAGTCTGAATACAAATCAGTCAAAACTTTCAACAATGGATCTCTTGGTTCCGGCATCGATGAAGAAC

GCAGCGAACTGCGATAAGTAATGTGAATTGCAGAATTCAGTGAATCATCGAGTCTTTGAACGCACATTGC

GCCCCCTGGCATTCCGGGGGGCATGCCTGTCCGAGCGTCATTGCTGCCCTTCAAGCCCGGCTTGTGTGTT

GGGTCGTCGTCCCCCTCGGGGGACGGGCCCGAAAGGCAGCGGCGGCACCGTGTCCGGTCCTCGAGCGTAT

GGGGCTTTGTCACCCGCTCGATTAGGACCGGCCGGGCGCCAGCCGGCGTCTCCAACCTTATTTTTCTCAG

GTTGACCTCGGATCAGGTAGGGATACCCGCTGAACTTAAGCATATCAATAAGCGGAGGAAAAGAAACCAA

CCGGGATTGCCTCAGTAACGGCGAGTGAA

>Aspergillus_sydowii_YFep2_(PV798564)

GCGGAAGGATCATTACTGAGTGCGGGCTGCCTCCGGGCGCCCAACCTCCCACCCGTGAATACCTAACACTG
TTGCTTCGGCGGGGAACCCCCTCGGGGGCGAGCCGCCGGGGACTACTGAACTTCATGCCTGAGAGTGATGC
AGTCTGAGTCTGAATATAAAATCAGTCAAAACTTTCAACAATGGATCTCTTGGTTCCGGCATCGATGAAGA
ACGCAGCGAACTGCGATAAGTAATGTGAATTGCAGAATTCAGTGAATCATCGAGTCTTTGAACGCACATTG
CGCCCCCTGGCATTCCGGGGGGCATGCCTGTCCGAGCGTCATTGCTGCCCATCAAGCCCGGCTTGTGTGTTG
GGTCGTCGTCCCCCCCGGGGGACGGGCCCGAAAGGCAGCGGCGGCACCGTGTCCGGTCCTCGAGCGTATG
GGGCTTTGTCACCCGCTCGACTAGGGCCGGCCGGGCGCCAGCCGACGTCTCCAACCATTTTTCTTCAGGTTG
ACCTCGGATCAGGTAGGGATACCCGCTGAACTTAAGCATAT

>Aspergillus_sydowii_YFep3_(PV798565)

CCTCCGGGCGCCCAACCTCCCACCCGTGAATACCTAACACTGTTGCTTCGGCGGGGAACCCCCTCGGGGGC
GAGCCGCCGGGGACTACTGAACTTCATGCCTGAGAGTGATGCAGTCTGAGTCTGAATATAAAATCAGTCAA
AACTTTCAACAATGGATCTCTTGGTTCCGGCATCGATGAAGAACGCAGCGAACTGCGATAAGTAATGTGAA
TTGCAGAATTCAGTGAATCATCGAGTCTTTGAACGCACATTGCGCCCCCTGGCATTCCGGGGGGCATGCCT
GTCCGAGCGTCATTGCTGCCCATCAAGCCCGGCTTGTGTGTTGGGTCGTCGTCCCCCCCGGGGGACGGGCC
CGAAAGGCAGCGGCGGCACCGTGTCCGGTCC
TCGAGCGTATGGGGCTTTGTCACCCGCTCGACTAGGGCCGGCCGGGCGCCAGCCGACGTCTCCAACCATTT
CTTCAGGTGAC

>Talaromyces_stollii_CBS_408.93_(NR_111781.1)

AAGGATCATTACCGAGTGCGGGCCCCTCGTGGCCCAACCTCCCACCCTTGTCTCTATACACCTGTTGCTT

TGGCGGGCCCACCGGGGCCACCTGGTCGCCGGGGGACGTTCGTCCCCGGGCCCGCGCCCGCCGAAGCGCT

CTGTGAACCCTGATGAAGATGGGCTGTCTGAGTACTATGAAAATTGTCAAAACTTTCAACAATGGATCTC

TTGGTTCCGGCATCGATGAAGAACGCAGCGAAATGCGATAAGTAATGTGAATTGCAGAATTCCGTGAATC

ATCGAATCTTTGAACGCACATTGCGCCCCCTGGCATTCCGGGGGGCATGCCTGTCCGAGCGTCATTTCTG

CCCTCAAGCACGGCTTGTGTGTTGGGTGCGGTCCCCCCGGGGGCCTGCCCGAAAGGCAGCGGCGACGTCC
GTCTGGTCCTCGAGCGTATGGGGCTTTGTCACTCGCTCGGGAAGGACTGGCGGGGGTTGGTCACCACCAC
AAAATTTTACCACGGTTGACCTCGGATCAGGTAGGAGTTACCCGCTGAACTTAAGCATATCAATAAGCGG
AGGAAAAGAAACCAACCGGG
